# Pathobiology of the 129:*Stat1*^−/−^ mouse model of human age-related ER-positive breast cancer with an immune infiltrate-excluded phenotype

**DOI:** 10.1186/s13058-017-0892-8

**Published:** 2017-09-02

**Authors:** Hidetoshi Mori, Jane Q. Chen, Robert D. Cardiff, Zsófia Pénzváltó, Neil E. Hubbard, Louis Schuetter, Russell C. Hovey, Josephine F. Trott, Alexander D. Borowsky

**Affiliations:** 1 Center for Comparative Medicine, University of California at Davis, Davis, CA USA; 2 Department of Pathology and Laboratory Medicine, School of Medicine, University of California at Davis, Sacramento, CA USA; 3 Department of Animal Science, University of California at Davis, Davis, CA USA

**Keywords:** Estrogen receptor, Luminal breast cancer, *Stat1*-knockout mouse, Pathobiology, Tumor microenvironment, Tumor immunology

## Abstract

**Background:**

*Stat1* gene-targeted knockout mice (129S6/SvEvTac-*Stat1* ^tm1Rds^) develop estrogen receptor-positive (ER^+^), luminal-type mammary carcinomas at an advanced age. There is evidence for both host environment as well as tumor cell-intrinsic mechanisms to initiate tumorigenesis in this model. In this report, we summarize details of the systemic and mammary pathology at preneoplastic and tumor-bearing time points. In addition, we investigate tumor progression in the 129:*Stat1* ^−/−^ host compared with wild-type 129/SvEv, and we describe the immune cell reaction to the tumors.

**Methods:**

Mice housed and treated according to National Institutes of Health guidelines and Institutional Animal Care and Use Committee-approved methods were evaluated by histopathology, and their tissues were subjected to immunohistochemistry with computer-assisted quantitative image analysis. Tumor cell culture and conditioned media from cell culture were used to perform macrophage (RAW264.7) cell migration assays, including the 129:*Stat1* ^−/−^-derived SSM2 cells as well as control Met1 and NDL tumor cells and EpH4 normal cells.

**Results:**

Tumorigenesis in 129:*Stat1* ^−/−^ originates from a population of FoxA1^+^ large oval pale cells that initially appear and accumulate along the mammary ducts in segments or regions of the gland prior to giving rise to mammary intraepithelial neoplasias. Progression to invasive carcinoma is accompanied by a marked local stromal and immune cell response composed predominantly of T cells and macrophages. In conditioned media experiments, cells derived from 129:*Stat1* ^−/−^ tumors secrete both chemoattractant and chemoinhibitory factors, with greater attraction in the extracellular vesicular fraction and inhibition in the soluble fraction. The result appears to be recruitment of the immune reaction to the periphery of the tumor, with exclusion of immune cell infiltration into the tumor.

**Conclusions:**

129:*Stat1* ^−/−^ is a unique model for studying the critical origins and risk reduction strategies in age-related ER^+^ breast cancer. In addition, it can be used in preclinical trials of hormonal and targeted therapies as well as immunotherapies.

**Electronic supplementary material:**

The online version of this article (doi:10.1186/s13058-017-0892-8) contains supplementary material, which is available to authorized users.

## Background

Mammary tumors in the 129/SvEv mouse strain with knockout (KO) of *Stat1* (129S6/SvEvTac-*Stat1* ^tm1Rds^; 129:*Stat1* ^−/−^ or *Stat1-*null) are unique among genetically modified mouse (GMM) models of human breast cancer [1]. In contrast to most other mouse models of human breast cancer, the resulting tumors are ovary-dependent and have uniformly high levels of estrogen receptor (ER)-α and progesterone receptor (PR), as well as elevated transcription of Forkhead box A1 (*FoxA1*) [2]. FoxA1-positive cells in the mammary gland are known to originate in the mammary ducts rather than in the lobuloalveolar units [3] and thus could potentially give rise to ductal tumors rather than alveolus-based tumors that occur in most mouse models of mammary cancer [4–6]. Further, *Stat1-*null females exhibit a prolonged tumor latency that models the majority of human breast cancers that are age-related [2, 7, 8]. Given these relevant characteristics, we sought to investigate the origins, evolution, and progression of tumors in the context of the whole animal, with detailed observations afforded by anatomic, histologic, endocrine function, immune cell reaction, and molecular evaluations of organs from aged 129:*Stat1* ^−/−^ mice [9–12].

The 129:*Stat1* ^−/−^ model has been used to study a variety of phenomena, including natural killer cell responses during obligate intracellular pathogen infection, immune editing, mammary gland development [2, 7, 13–15], and mammary tumorigenesis [2]. Regarding changes in the mammary glands, these studies documented deficiencies in the immune and endocrine systems; dramatic changes in cytokine concentrations both systemic and tissue; localized effects on mammary development and the microenvironment; and the spontaneous, cell-autonomous development of mammary neoplasia [2, 16]. Reciprocal mammary gland transplantation in immune intact syngeneic 129/SvEv and in 129:*Stat1* ^−/−^ mice, as well as tissue coculture experiments, has facilitated isolation and investigation of the host environment [7].

The luminal mammary carcinomas in 129:*Stat1* ^−/−^ occur as spontaneous neoplasms in nulliparous and parous females, and they have a prolonged latency [2]. The tumor cytology is unique among GMM models of mammary cancer and is relatively uniform throughout all tumors. Molecular analysis of *Stat1*-null tumors revealed decreased levels of Janus kinase 2 (JAK2) and downstream activity resulting in tumorigenesis [2, 16]. Moreover, genomic analysis of mammary tumors arising in the 129:*Stat1* ^−/−^ mouse established they almost always harbored a truncating exon 10 mutation of the prolactin receptor (PRLR) that was tumorigenic when heterozygously expressed in embryonic fibroblasts lacking *Stat1* but overexpressing *Jak2* [17].

Although mammary gland development in the 129:*Stat1* ^−/−^ model was initially described as normal [2], a subsequent and more detailed study revealed that the mammary glands had delayed development, defective branching morphogenesis, and abnormal terminal end buds [7]. Further, the gland-free KO mammary fat pad produces only low concentrations of a number of growth factors/cytokines [7]. Interestingly, mammary growth and the cytokine profile can be restored to normal levels either with pregnancy or by administering exogenous progesterone and prolactin (PRL) [7]. Therefore, the 129:*Stat1* ^−/−^ model provides an opportunity to investigate the dependence of mammary development and tumorigenesis on endocrine-cytokine interactions between the tumor and the microenvironment.

KO of *Stat1* in other mouse strains and using other targeting approaches does not lead to spontaneous primary mammary tumors. When these *Stat1* ^−/−^ mice are crossed with *Neu*-expressing GMM, the resulting bigenic animals do, however, exhibit accelerated tumorigenesis, suggesting that STAT1 functions as a tumor suppressor [18–21]. Forced breeding of Balb/c:*Stat1* ^*−/−*^ females stimulated mammary neoplasia but resulted in a variety of tumor types that are different from the homogeneous 129:*Stat1* ^*−/−*^ tumors [21]. Thus, the 129:*Stat1* ^−/−^ model offers the unique opportunity to identify host and tumor factors modeling similar human breast cancers [2, 17]. Mammary tumors in 129:*Stat1* ^−/−^ occur exclusively in aged females and depend on hormonal and microenvironmental changes. Their ER^+^ luminal phenotype, which is the most common phenotype in human breast cancers and the most common phenotype associated with advanced age, occurs only in *Stat1* KO on the 129 background, implying there are genetic modifiers of this phenotype.

We have studied the origin and progression of primary mammary tumors in 129:*Stat1* ^*−/−*^ in our colony from early development [7] and, now, to over 2 years of age (120 weeks). We report the findings of additional morphological abnormalities in the *Stat1* ^−/−^ mouse that reflect systemic endocrine and environment effects, as well as the identification of a FoxA1^+^ large oval pale (LOP) cell that appears along some *Stat1*-null ducts as their hosts approach tumor-bearing age. These LOP cells precede the invasive cancers, and their distribution is consistent with evolution of a mutant mammary epithelial cell (MEC) clone that populates contiguous segments of the mammary gland.

**Fig. 1:**
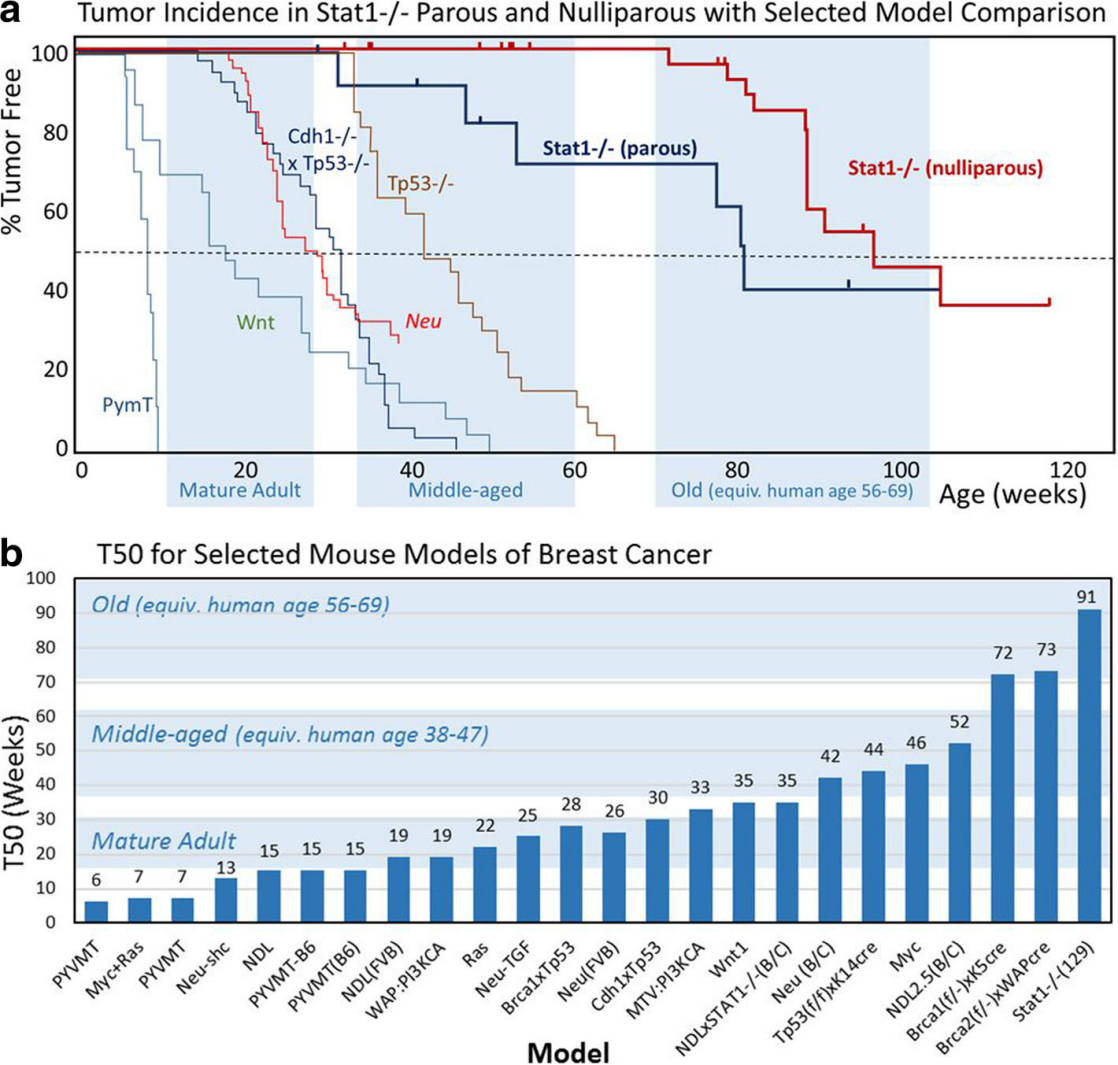
Kaplan-Meier and *T*_50_ plots comparing tumorigenesis in 129:*Stat1* ^−/−^ and other published models. **a** Kaplan-Meier plots showing survival curves for parous and nulliparous 129:*Stat1* ^−/−^ as compared with those from the *Wnt*, *Neu*, *PyVMT*, *Tp53* ^−/−^, and *Cdh1* ^−/−^/*Tp53* ^−/−^ models. The *blue highlighting* indicates the ages in weeks when mice are considered mature, middle-aged, and old adults as calculated by Harrison’s laboratory [44]. **b** Chart of the *T*_50_ values for 129:*Stat1* ^−/−^ and a variety of common mouse models of human breast cancer. Where the *T*_50_ was not provided, the median value was used as the *T*_50_. The *blue highlighting* indicates the ages in weeks during which mice are considered mature, middle-aged, and old adults [44]

A role for the microenvironment in the initiation of cancers is clear, given the age dependency of the cancers and their thoracic location. We also describe an unusually strong host immune response (compared with other GMM mammary cancer models, including GMM transplants such as the Met1 model Fig. 1a [22]) to the invasive *Stat1*-null neoplasm that increases with lesion progression. These host responses are similar in both 129:wild-type (129:WT) and 129:*Stat1* ^*−/−*^ hosts, suggesting that the tumor cells produce chemoeffectors, which were subsequently found in both the soluble and the exosome fractions from cultured tumor cells.

## Methods

### Mouse model

129:*Stat1* ^−/−^ mice [7, 23] were provided by the Schreiber laboratory (Washington University, St. Louis, MO, USA), and 129S6/SvEvTac mice were purchased from Taconic Farms (Hudson, NY, USA). Mice were housed in a vivarium according to National Institutes of Health guidelines, and all animal experiments were performed following procedures approved by the UC Davis Institutional Animal Care and Use Committee. The procedures for transplantation [7] and the conditions for maintaining mice [24] were described previously.

### Histopathology and whole-mount preparation

Most tissues were fixed in 10% neutral buffered formalin (NBF) at room temperature for 24 h, then placed in 70% ethanol until processing, which was normally within 24 h. To validate the immunohistochemical staining with NBF-fixed samples, some tissues were fixed in zinc-based fixative as described elsewhere [24]. Procedures for infiltrating tissue with paraffin, sectioning, and hematoxylin and eosin (H&E) staining were described previously [24]. The preparation of whole mounts of mouse mammary glands was carried out as previously described [7].

### Transplants

Fragments (1 mm^3^) of freshly dissected tumors were transplanted into uncleared thoracic and inguinal mammary fat pads of 3- to 6-week old nulliparous WT and *Stat1*-null females under appropriate anesthesia and sterile conditions as previously described [7]. The animals were monitored at least twice per week. Growth of palpable tumors was noted and measured in two dimensions using calipers. The volumes were calculated using the average diameter [4/3 × π × (d1 + d1/4)^3^].

### Immunohistochemistry

Immunohistochemistry (IHC) was performed as previously described [7, 24]. Stained slides were scanned on an Aperio AT2 ScanScope (Leica Biosystems, Buffalo Grove, IL, USA), and digital images were viewed using the ImageScope application (Leica Biosystems). Digital images were captured and processed using Photoshop software (Adobe Systems, San Jose, CA, USA). The imaging analysis for counting marker-positive cells was performed with inForm Cell Analysis software (PerkinElmer, Waltham, MA, USA). Multiplex IHC was performed with tyramide signal amplification (TSA)-based fluorescence color visualization [25]. TSA-based multiplex IHC was performed according to the manufacturer’s protocol (PerkinElmer). FoxA1, epithelial cell adhesion molecule (EpCAM), and keratin-14 (KRT14) were detected with TSA Plus fluorescein, cyanine 3 (Cy3), and Cy5, respectively. Visualization of multiplexed images was performed with an LSM710 laser scanning confocal microscope (Carl Zeiss Microscopy, Oberkochen, Germany). Antibodies used were anti-ER-α (clone MC-20; Santa Cruz Biotechnology, Dallas, TX, USA), anti-PR (polyclonal; Dako North America, Carpinteria, CA, USA), anti-Ki67 (clone Ab-4, Lab Vision; Thermo Fisher Scientific, Fremont, CA, USA), anti-CD3 (clone SP7; Abcam, Cambridge, MA, USA), anti-CD4 (clone RM4-5; eBioscience, San Diego, CA, USA), anti-CD8a (clone 4SM15; eBioscience), anti-F4/80 (clone MCA497, AbD Serotec; Bio-Rad Laboratories, Hercules, CA, USA), anti-B220 (clone RA3-6B2; BD Biosciences, San Jose, CA, USA), anti-KRT14 (Covance Poly19053; BioLegend, San Diego, CA, USA), anti-FoxA1 (clone EPR10881; Abcam), anti-EpCAM (EPR677; Abcam), anti-KRT8/18 (CK209; Fitzgerald Industries International, Acton, MA, USA), and anti-type IV collagen (polyclonal; Abcam).

### Collecting conditioned media

A *Stat1*-null cell line (SSM2) [2], an *MMTV-NDL*-derived cell line (NDL) [26], and an *MMTV-PyMT*-derived cell line (Met1) [22] were cultured in T75 culture flasks at 1 × 10^6^ cells in growth medium (DMEM supplemented with 10% FBS) for 2 days. A phenotypically normal MEC line, EpH4, was cultured as described previously [27]. To prepare conditioned medium (CM) from each cell line, cells were rinsed three times with serum-free medium and then cultured in 10 ml of serum-free medium for 48 h. CM was collected, centrifuged to eliminate cells and debris, and filtered (0.2 μm). To isolate extracellular vesicles (EVs), CM was ultracentrifuged at 100,000 × *g* for 2 h. The pellet (EV-rich fraction) was resuspended in DMEM with the same volume as the supernatant (soluble fraction).

### Time-lapse migration assay

The green fluorescent protein (GFP)-tagged H2B [28] was transduced into RAW264.7 mouse macrophages (American Type Culture Collection, Manassas, VA, USA) using pLenti-EF1a-Puro bearing a GFP-tagged H2B complementary DNA [27]. Preparation of lentiviral particles and transduction of target cells were performed as previously described [27]. To maintain GFP-H2B-positive cells, growth medium was supplemented with 0.5 μg/ml puromycin. To observe RAW264.7/GFP-H2B cell migration, cells were plated at 2 × 10^4^ cells/well of an eight-well Lab-Tek II chambered coverglass (Thermo Fisher Scientific, Waltham, MA, USA) in growth medium for 2 days before stimulating cell migration with CM. Fluorescence was visualized with an LSM710 confocal microscope equipped with a temperature- and CO_2_-controlled chamber [29, 30]. Before cell migration was analyzed, cells were rinsed twice and maintained in 400 μl of serum-free DMEM for 2 h. The action of RAW264.7/GFP-H2B cells was monitored at 5-minute intervals for more than 8 h. Cell migration was evaluated using time-lapse images with Imaris software (Bitplane, South Windsor, CT, USA).

### Transwell migration assay

RAW264.7 cells were resuspended in DMEM at a density of 1 × 10^6^ cells/ml, and 100 μl of resuspended cells were placed into the upper chamber of Transwell culture inserts (8-μm pore size) in 24-well plates (Corning, Corning, NY, USA). Quantities of 600 μl of DMEM or CM from each cell line were applied in the bottom chamber for 5 h to test the chemoattractant activity. Cells on the underside of the insert were fixed with 70% ethanol for 10 minutes and then stained with 0.2% crystal violet before rinsing to remove background staining and air-drying, followed by microscopic imaging.

### Statistical analysis

All statistical analyses were done using Prism 7 software (GraphPad Software, La Jolla, CA, USA). Kaplan-Meier plots were generated to compare the tumorigenesis of nulliparous and multiparous 129:*Stat1* ^−/−^ animals (Fig. 1). The difference was detected with the log-rank test. Similarly, Kaplan-Meier plots were created to compare the latency of palpable tumor onset between tumors transplanted to 129:*Stat1* ^−/−^ or 129:WT hosts. The difference was detected with the log-rank test. The asymmetries of primary tumor occurrence between left-right and caudal-cephalad mammary fat pads (Additional file 1: Table S1) were tested with a binomial test.

## Results

### Tumor incidence and distribution

A total of 24 palpable mammary tumors developed in 20 female 129:*Stat1* ^−/−^ mice. Tumors were detected in two tumor-bearing parous females before 1 year of age (at 32 and 47 weeks). The other 18 tumor-bearing mice developed tumors between the ages of 1 year (52 weeks) and 2.3 years (120 weeks). For comparison, 30 female 129:*Stat1* ^−/−^ mice without palpable tumors were killed at various times beyond 52 weeks of age (Additional file 1: Table S2). The differences in time to tumor onset between parous and nulliparous females in our colony were significant (*p* = 0.038 by log-rank test), consistent with the initial report about the Washington University in St. Louis colony [2] that had an equally prolonged latency and incomplete (~53%) penetrance. Although the tumor incidence in our cohort remained consistent after 1 year of age (35%), subsequent pathological analysis revealed that surviving nulliparous females had mammary intraepithelial neoplasia (MIN) lesions, suggesting that the penetrance of mammary tumors could approach 100% were animals to age even further.

The 13 tumor-bearing nulliparous females ranged in age at tumor onset from 73 to 120 weeks, with 50% incidence at 91 weeks. The comparable 24 tumor-free nulliparous WT females covered a similar age range (53–120 weeks) and a similar average age when killed (83 weeks). Similar to a previous report [2], pregnancy shortened tumor latency from *T*_50_ = 91 weeks to *T*_50_ = 78 weeks. A comparable cohort of six tumor-free parous KO mice was examined (mean age 83 weeks, range 52–95 weeks). The fact that not all aged 129:*Stat1* ^−/−^ animals were tumor-bearing within 120 weeks also suggests a stochastic component to tumor initiation in addition to the GMM intrinsic susceptibility (Fig. 1).

When these data are compared with the recorded or calculated *T*_50_ from other GMM models that are KOs of tumor suppressor genes or employ a mammary-specific promoter driving the targeted expression of an oncogene, the 129:*Stat1* ^−/−^ model stands out as being unique (Fig. 1). The *T*_50_ for 129:*Stat1* ^−/−^ is over three times the mean *T*_50_ (28 weeks) of these other GMMs and 40 weeks longer than the closest model, *MMTV-cMyc* [31]. When compared with other GMMs using their respective Kaplan-Meier plots, the 129:*Stat1* ^−/−^ model plot does not overlap with any of those for commonly reported models (Fig. 1).

### Tumor topography

The palpable mammary tumors showed a notable cephalad (thoracic) to caudal (inguinal) dominance (Additional file 1: Table S1). Eighty-seven percent (21 of 24) of tumors were detected in the thoracic mammary fat pads (*p* = 0.0003 by binomial test) (Additional file 1: Table S1). These distributions are consistent with the topographical asymmetries previously described in other models and suggest that the local microenvironment influences tumor development [32]. In addition, the majority of palpable tumors were proximate to the nipple rather than at the periphery of the fat pad. These masses around or adjacent to the nipple can also explain the dilation and congestion of some more distal ducts (Additional file 2: Figure S1).

### Necropsy pathology

**Fig. 2:**
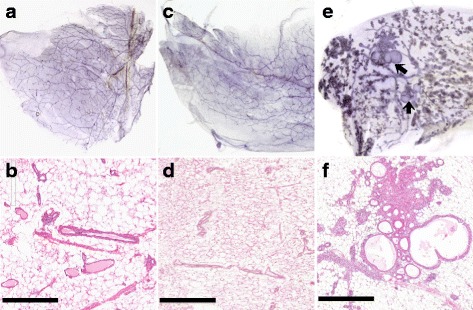
Normal and diseased 129:*Stat1* ^*−/−*^ mammary glands with mammary intraepithelial neoplasia (MIN). This figure compares representative mammary whole mounts and representative hematoxylin and eosin (H&E)-stained histology for (**a** and **b**) an 88-week-old nulliparous 129:wild type (129:WT), (**c** and **d**) tumor-free 129:*Stat1* ^*−/−*^, and (**e** and **f**) 120-week-old nulliparous tumor-bearing 129:*Stat1* ^*−/−*^ *.* The tumor-free knockout and WT are normal (**a**–**d**). The whole mount from the nulliparous, tumor-bearing, 120-week-old 129:*Stat1* ^*−/−*^ female shows extensive lobuloalveolar development and two cystic MIN (*arrows*) (**e**). The H&E staining shows cystic lesions and a dense duct without a visible lumen filled with precancerous large oval pale cells (MIN) (**f**). Scale bars = 500 μm

**Fig. 3:**
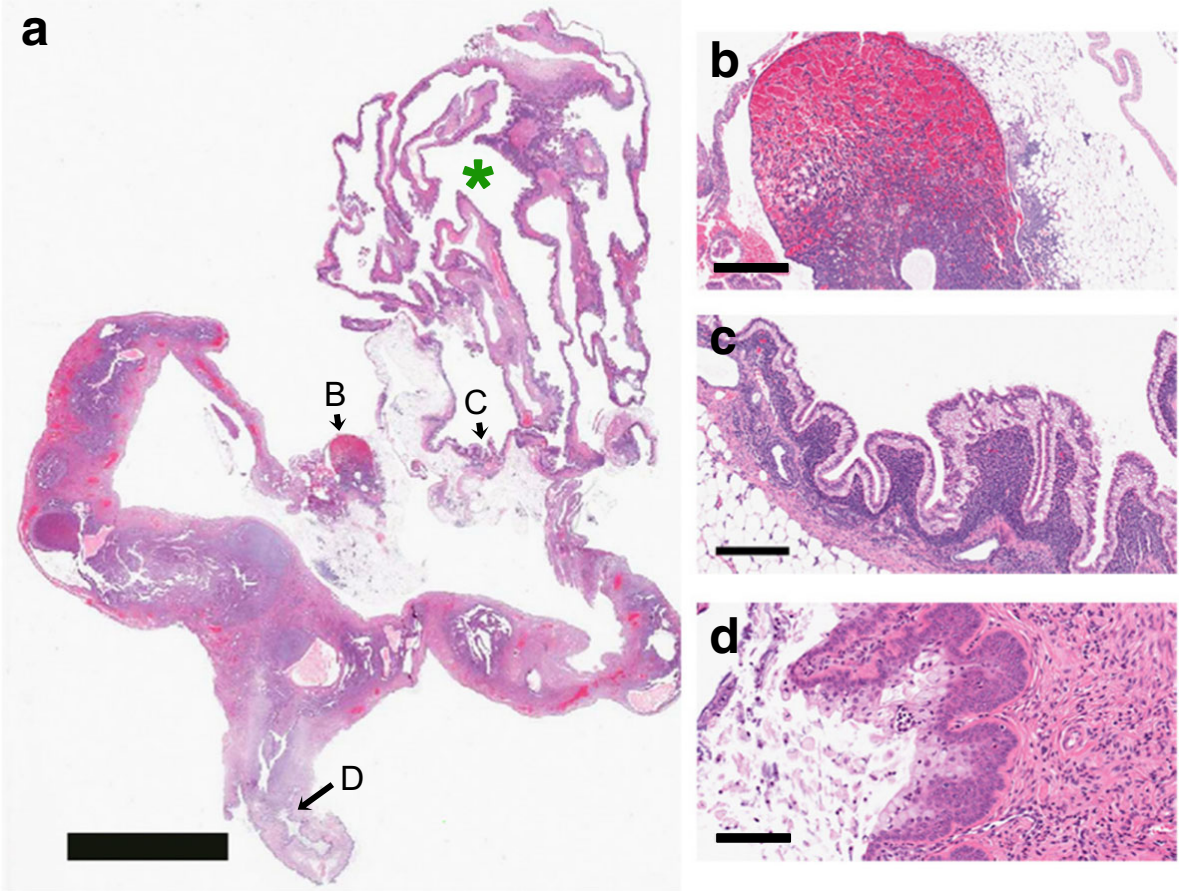
Observations in knockout gynecology. This figure illustrates the ovaries, uterus, and vagina of a 120-week-old 129:*Stat1* ^−/−^nulliparous female. **a** This low-magnification image shows that the right ovary is obliterated by a large cyst (*green asterisk*). *Arrows B*, *C*, and *D* indicate regions of interest for the higher-magnification images shown in **b**, **c**, and **d**, respectively. Scale bar = 5 mm. **b** The contralateral ovary is largely replaced by multiple vascular channels filled with red blood cells. Scale bar = 400 μm. **c** The cyst is lined with a tall columnar epithelium with apical nuclei characteristic of rete cysts of the mouse ovaries. Scale bar = 200 μm. **d** In spite of the destruction of the ovaries, the vaginal surface has a layer of bluish mucinous cells associated with proestrus. This indicates a functional estrous cycle. Scale bar = 100 μm

Necropsy of the three subcohorts (129:WT, tumor-free 129:*Stat1* ^*−/−*^, and tumor-bearing 129:*Stat1* ^*−/−*^) was performed with attention to microscopic mammary and nonmammary pathologies that may correlate with the tumor-bearing phenotype (Figs. 2 and 3). The older animals in all three cohorts shared sporadic conditions associated with aging, such as bronchioloalveolar adenoma, eosinophilic (crystalline) pneumonitis, atrophic cystic hyperplasia of the endometrium, segmental interstitial nephritis, ovaries with luteinized stroma and fewer Graafian follicles, and low-grade small cell lymphoma [33]. The adrenal glands, thyroid glands, pancreas, and pituitary glands were all disease-free. Significantly, the vaginal mucosa in all three cohorts had evidence of ovarian function at older ages. For example, at 96 weeks of age, the vaginal mucosa of both KO and WT displayed cornification (estrus), inflammation (metestrus), or mucinous differentiation (proestrus). The vaginal mucosa is a standard histological indication of stage of the estrous cycle (*see* Fig. 3). In contrast, the mammary glands and ovaries had features unique to each cohort, as described below (Figs. 2 and 3).

#### 129:WT

Two 129:WT females were held until 97 weeks. One female was parous and had eosinophilic pneumonitis, a polycystic nonproliferative endometrium, and luteinized ovarian stroma with scattered follicles. One ovarian bursa was cystically dilated. The mammary glands had mild lobuloalveolar development with scattered inflammatory (squamous) nodules, consistent with persistent postinvolutional hyperplasia [34]. The mammary glands of the nulliparous 97-week-old female were devoid of inflammatory nodules and hyperplasia. The uterus and ovaries of the two mice were similar in that they had a cystic endometrium and luteinized ovarian stroma with reduced follicles.

#### 129:*Stat1*^*−/−*^

The 129:*Stat1* ^*−/−*^ mice younger than 32 weeks old (*n* = 42) did not have detectable histopathology in any of their organs. The mammary glands of non-tumor-bearing 129:*Stat1* ^*−/−*^ females that were aged 52 weeks or older (*n* = 32) were also examined. Of the older nulliparous females without tumors, three had microscopic MIN in their mammary glands (Fig. 2). In contrast, the mammary glands of fifteen of the twenty-three 129:*Stat1* ^*−/−*^ females bearing preneoplastic MIN or tumors had various hyperplastic and dysplastic features (Fig. 2e, f).

Mammary glands in 12 of the 20 tumor-bearing animals also had lobuloalveolar hyperplasia that sometimes obscured the MIN at the gross examination level. The remaining eight females had sparsely branched mammary ductal networks. The lobuloalveolar hyperplasia in 9 of 13 nulliparous tumor-bearing females is noteworthy because these animals had not been pregnant or exposed to the associated hormonal environment. In addition, most of these animals had dilated mammary ducts filled with proteinaceous “milky” white fluid (Additional file 2: Figure S1). The heterogeneity of mammary development was striking where different levels and patterns of lobuloalveolar development were present in the same mammary gland (Fig. 2e). The other four nulliparous tumor-bearing females had underdeveloped and stunted mammary ductal networks that failed to fill the fat pad [7].

#### Ovaries

All 129:*Stat1* ^*−/−*^ females older than 1 year of age had periovarian or ovarian tubules identified as mesonephric or rete tubules [35, 36] (Fig. 3a). In addition, thirty-one of the fifty 129:*Stat1* ^*−/−*^ females had ovaries with multiple cysts, some of which completely ablated the ovaries (Fig. 3b). Most cysts were lined by a simple attenuated epithelium. However, many ovarian cysts were lined by a tall columnar epithelium with apical nuclei signifying them as rete cysts (Fig. 3c) [36]. Notably, in this specific case, the contralateral ovary was obliterated by a highly vascular hemorrhagic mass (Fig. 3d). However, the vagina had a mucinous epithelium indicative of proestrus and continued estrous cyclicity (Fig. 3d). One 84-week-old tumor-bearing female had histologically normal ovaries with multiple Graafian follicles. Ovaries without cysts showed luteinized stroma and reduced germinal follicles. Therefore, tumorigenesis did occur in the absence of ovarian cysts, and not all mice/females with ovarian cysts developed mammary tumors.

#### Uterus

The uterus of 129:*Stat1* ^*−/−*^ mice tended to be hypervascular, where five females had dense tangles of vessels that were classified as hemangiomas. These uteri had frequent dilated vessels with blood and fibrin thrombi. One tumor-bearing animal had a stromal sarcoma of the uterus, and another had an endometrial focus consistent with adenocarcinoma.

### Origin and evolution of *Stat1*-null tumors

**Fig. 4:**
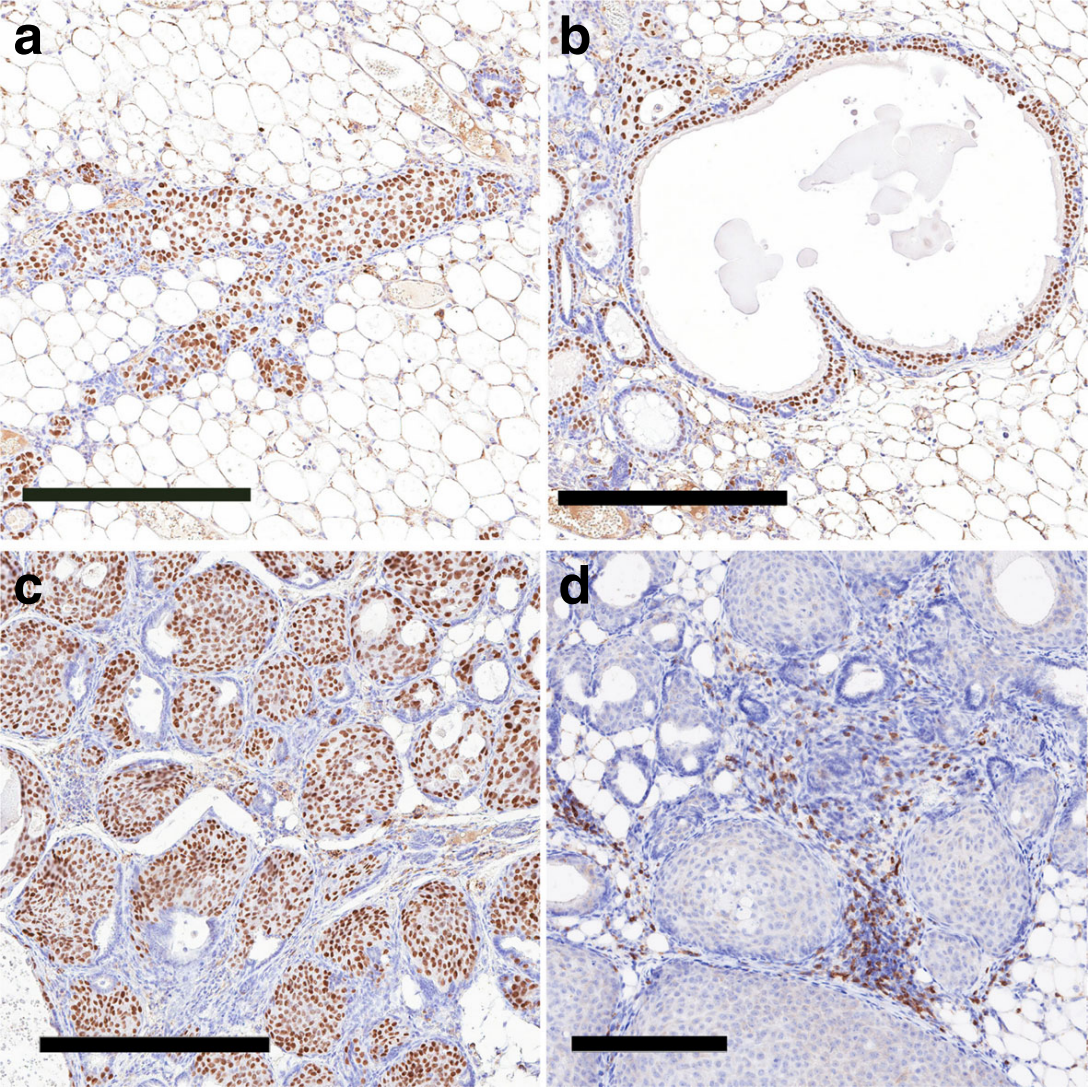
Histological types of mammary intraepithelial neoplasia (MIN). **a**–**d** This figure illustrates the three types of MIN lesions found in a 129:*Stat1* ^*−/−*^ female, stained for Forkhead box A1 (**a**–**c**; 106-week-old nulliparous female) and CD3 (**d**; 90-week-old tumor-bearing female). **a** Ductal pattern with a branching duct filled with large oval pale (LOP) cells. Scale bar = 300 μm. **b** Cystic with expanded luminal cystic space lined by LOP cells. Note the lack of any appreciable host response in **a** and **b**. Scale bar = 300 μm. **c** Solid micronodular pattern with small nests of cells in dense connective tissue. Scale bar = 200 μm. **d** A higher-magnification image of a more advanced solid nodular MIN showing dilated vessels and dense round cell infiltrate with CD3^+^ T cells (*see* Fig. 9). Scale bar = 100 μm

The early, preinvasive MIN lesions in 129:*Stat1* ^−/−^ females have been mentioned previously without morphologic descriptions [2, 17]. These lesions are atypical foci that stand out from the surrounding normal mammary epithelium but remain confined within the basement membrane (Figs. 2 and 4), thus fitting the morphological criteria for MIN [4, 37]. These MIN could be found contiguous or adjacent to invasive tumors as well as in nontumorous glands and ducts of 129:*Stat1* ^−/−^ tumor-bearing females, as well as in some tumor-free females older than 52 weeks. These MIN never occurred in 129:WT females, including two 97-week-old females, or in disease-free 129:*Stat1* ^−/−^ females younger than 1 year of age (*n* = 42).

**Fig. 5:**
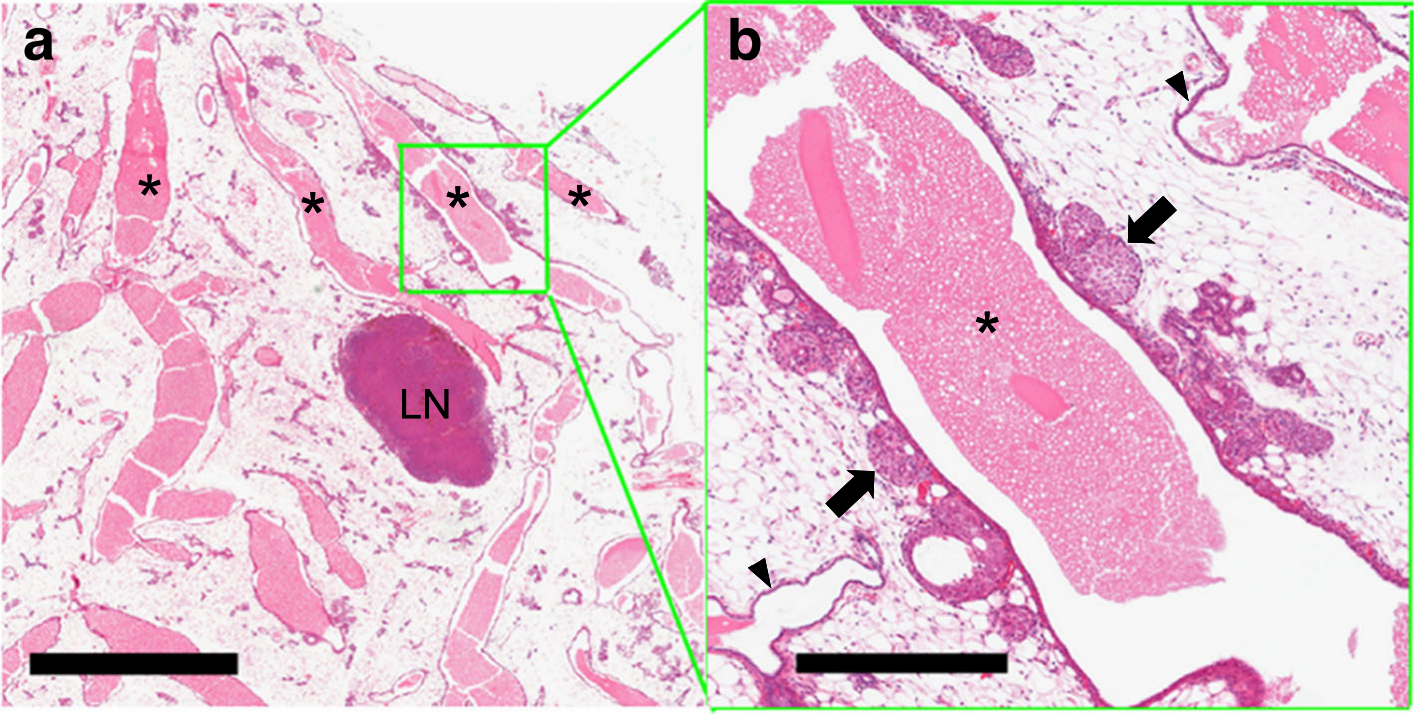
The diseased duct. A low-magnification view of the mammary gland from the tumor-free right inguinal (#4) mammary fat pad of a nulliparous 106-week-old tumor-bearing 129:*Stat1* ^*−/−*^ female showing four engorged ducts (*asterisks*) coursing through the mammary fat pad (**a**). *LN* Lymph node. Scale bar = 2 mm. Note that the large oval pale (LOP) cell-forming multiple aberrant side buds are restricted to one duct (*green box*) (**b**) and have a nodular profile (*arrows*). Other main stem ducts and their branches have smooth outlines and a normal basal and luminal bilayered epithelium (*arrowheads*). In addition, Additional file 2: Figure S3 shows quantitation of the distribution of LOP cells. The DNA sequences of laser capture microdissection from the diseased duct within the *green box* showed three different single-nucleotide variations in the prolactin receptor (PRLR) gene. A higher-magnification image shows the solid abortive side buds filled with LOP cells of the diseased duct. Scale bar = 400 μm. Figure 6c shows a higher-magnification view of progesterone receptor-stained LOP cells in the solid nodules of the same duct

The *Stat1*-null MIN are small, nonpalpable, and not easily identified by gross inspection. Obvious lesions can be identified at the subgross level in mammary gland whole mounts as discrete, hypercellular foci that stand out from the background, typically as solid ducts, micronodular clusters, or small cysts (Fig. 4, Additional file 2: Figure S2e–g). The smaller MIN were not associated with a significant host response. The larger, presumably more advanced lesions were surrounded by a significant host inflammatory response that had a rich mast cell component but were composed primarily of T cells, macrophages, and increased vascularity (Fig. 4d, Additional file 2: Figure S2h). The distribution of MIN in affected glands was restricted to one or several ducts of the mammary network, whereas adjacent ducts had normal histology and cytology (Fig. 5a, b).

**Fig. 6:**
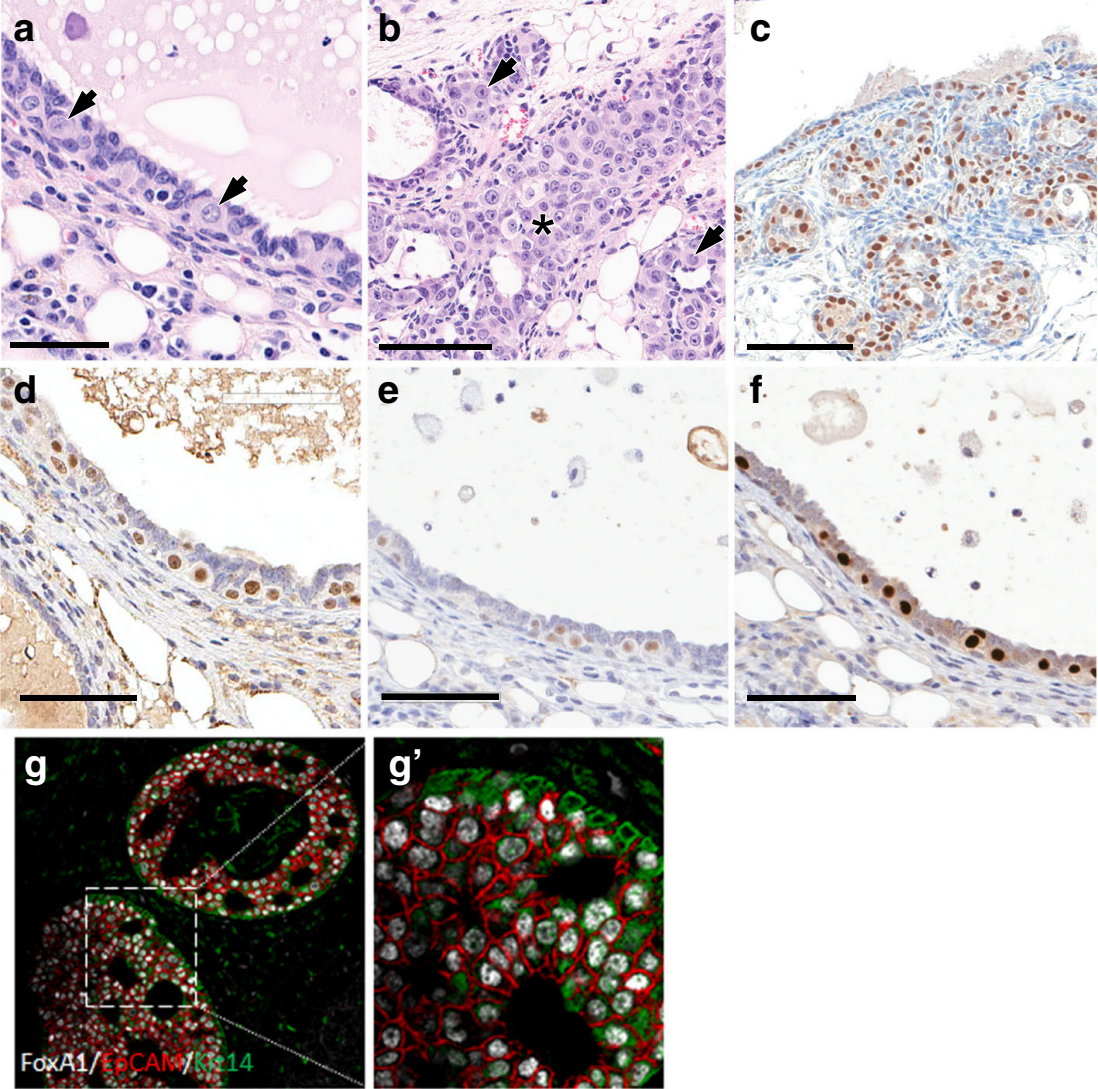
Large oval pale (LOP) cells in 129:*Stat1* ^*−/−*^ mammary glands developing cancer. The figure illustrates the potential cancer-initiating LOP cells in 129:*Stat1* ^*−/−*^ mammary intraepithelial neoplasia (MIN) and tumors. The LOP cell has a pale cytoplasm and a large oval nucleus with clear chromatin. These cells initially appear in ducts before MIN are detectable. **a** Hematoxylin and eosin (H&E) staining of duct shows LOP cells standing out from the normal luminal layer (*arrows*). The normal mammary epithelial cells (MECs) have densely staining nuclei and relatively sparse cytoplasm. These LOP cells were found only in aging 129:*Stat1* ^*−/−*^ females with tumors or MIN. Scale bar = 100 μm. **b** H&E-stained image shows LOP cells filling a duct (*asterisk*) and populating side buds (*arrows*). Scale bar = 100 μm. **c** Progesterone receptor (PR)-stained tissue shows side budding that is forming an early MIN lesion. Scale bar = 70 μm. **d** Forkhead box A1 (FoxA1)-positive LOP cells were observed in a duct from a tumor-bearing mouse. An adjacent duct was negative for FoxA1. Scale bar = 70 μm. **e** and **f** Immunohistochemical (IHC) stains show uniformly positive LOP cells for (**e**) estrogen receptor (ER) and (**f**) PR. FoxA1^+^/ER^+^/PR^+^ LOP cells in tumor are shown in Additional file 2: Figure S6. **g** and **g**′ A multiplex IHC image shows FoxA1 (*white*), epithelial cell adhesion molecule (*red*), and keratin 14 (*green*) expression in MIN in 129:*Stat1* ^*−/−*^. **g**′ Higher-magnification image of *inset* shown in **g**. Note the dual staining for basal (*green*) and luminal (*red*) antigens in many of the LOP cells, indicating that they are dual-staining and potentially pluripotential

Microscopically, the MIN were characterized by clusters of unique atypical cells that stood out from the adjacent MEC. These dysplastic cells had large, oval, moderately pleomorphic nuclei with an open chromatin pattern and prominent nucleoli with abundant pale cytoplasm (LOP cells) (Fig. 6a, Additional file 2: Figure S3). These LOP cells are large relative to adjacent luminal and basal MECs (Fig. 6, Additional file 2: Figure S3). In addition, LOP cells in MIN and associated malignancies were KRT8/18^+^, with some also being KRT14^+^ but negative for smooth muscle actin (Additional file 2: Figure S4). The LOP cells were further characterized using multiplexed immunofluorescence, which revealed nuclear staining for FoxA1 in neoplastic EpCAM^+^ luminal cells and in sparse populations of KRT14^+^ basal-like cells, suggesting that FoxA1^+^ cells within MIN might be pluripotent (Fig. 6g, g′). The cells in *Stat1*-null invasive carcinomas (tumors) shared these immunophenotypic characteristics.

Neoplasms *in Stat1*-null mice contained LOP cells within MIN (Figure 6b, c; Additional file 2: Figure S5). These LOP cells were also interspersed among normal ductal MECs along relatively normal ducts (Fig. 6a, b, and d–f; Additional file 2: Figure S5). In general, the LOP cells appear to be more basally oriented but remained inside the myoepithelial layer (suprabasal). In some presumably more advanced cases, small collections of LOP cells appeared as multicellular nodules along the ducts (Figs. 5 and 6). In other instances, these LOP cell nodules formed outgrowths attached to but bulging out from the main duct (Fig. 5, Additional file 2: Figure S1). These nodules resembled abortive ductal side buds in normal mammary glands.

The distribution of LOP cells and MIN was restricted to one or several ducts of the mammary network in affected glands (Fig. 5), whereas the other adjacent ducts had an entirely normal histology and associated cytology (Fig. 5). The LOP cells in the “diseased” duct were FoxA1^+^, ER^+^, and PR^+^ (Fig. 6, Additional file 2: Figure S6). Further, laser capture-microdissected samples from the diseased duct illustrated in Fig. 5 were extracted, and exon 10 of the PRLR was sequenced. The specific duct (right inguinal mammary gland) contained three separate nontruncating single-nucleotide variants. In contrast, the adjacent ducts and lymph node had a normal WT PRLR sequence, whereas the palpable mammary tumor in the same animal’s left thoracic mammary gland was heterozygous for a truncating PRLR mutation similar to those previously described [17].

### Tumor microscopic description

The microscopic characteristics of the 129:*Stat1* ^*−/−*^ tumors have been described previously and display consistently similar morphologic phenotypes from tumors in one mouse to tumors in the next (low intertumoral heterogeneity) [2]. In contrast, spontaneous tumors from *Trp53*- and *Brca1*-KO mice, as well as from pregnancy-induced Balb/c:*Stat1* ^−/−^ tumors, have a mixture of tumor types, with basal-like and “Wnt pathway” phenotypes being predominant [5, 21, 38]. Balb/c:*Stat1* ^−/−^ mice crossed with mice expressing other oncogenes develop tumors with phenotypes identical to those of the oncogenic transgene [18–20]. The signature 129:*Stat1* ^−/−^ tumor phenotype consists of nodular nests of cells with large oval nuclei and abundant pale cytoplasm. These cells are cytologically identical to the LOP cells found in MIN. Eighty percent to 95% of these cells in MIN and tumors were immunopositive for ER, PR, and FoxA1 (Fig. 4, Additional file 2: Figures S5 and S6).

#### Invasion and metastasis

In contrast to the expansile margins found in most mouse mammary tumors [5], *Stat1*-null tumors are characterized by local invasion into the surrounding tissues (Additional file 2: Figure S7a). Metastasis to local mammary lymph nodes was observed in three tumor-bearing KO mice (Additional file 2: Figure S7b). Occasional vascular invasion was also observed (Additional file 2: Figure S7c and d). However, only one tumor-bearing animal had micrometastases to the lung, which is the most common site of metastasis in GMM models [5]. There was no evidence of metastasis to other organs.

#### Test by transplantation

**Fig. 7:**
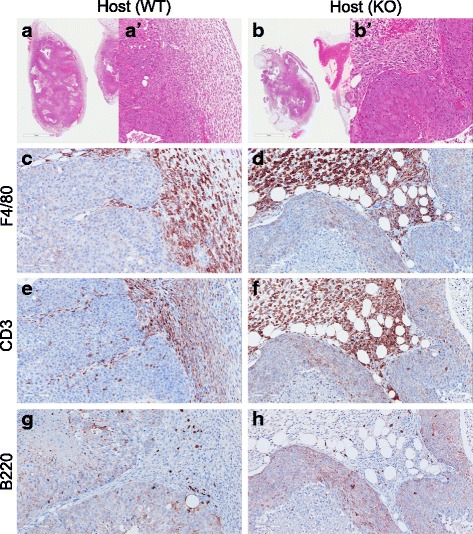
Immune cells in 129:*Stat1* ^*−/−*^ tumor transplants in fat pads of 129:wild-type (129:WT) and 129:*Stat1* ^*−/−*^ mice. Hematoxylin and eosin-stained tissue sections of tumor transplants grown in inguinal fat pads of (**a**, **a**′) 129:WT and (**b**, **b**′) 129:*Stat1* ^*−/−*^ mouse mammary fat pads. **a′** and **b′** Higher-magnification images of tumor-stroma boundaries in **a** and **b**. Also shown are (**c**, **d**) macrophages, (**e**, **f**) T lymphocytes, and (**g**, **h**) B lymphocytes detected in 129:*Stat1* ^*−/−*^ tumor transplants in (**c**, **e**, **g**) 129:WT and (**d**, **f**, **h**) 129:*Stat1* ^*−/−*^ mice. *KO* Knockout

A more detailed study of the 129:*Stat1* ^−/−^ neoplastic growth was performed by transplanting both primary MIN and tumor tissues. Multiple attempts to isolate and transplant MIN-type lesions in syngeneic WT mice failed to produce an outgrowth line. However, primary tumors were readily transplanted into the intact mammary glands of young WT female mice and into young, age-matched 129:*Stat1* ^*−/−*^ female mice (Fig. 7). Interestingly, transplants into young WT hosts became palpable and attained volume endpoints sooner than the same tumor transplanted into young 129:*Stat1* ^*−/−*^ hosts (*p* = 0.021 by log-rank test). Once tumors were palpable, their growth rates were similar (Additional file 2: Figure S8). Thus, when the experiments were terminated at 53 days, the tumor volumes in the WT hosts were 10 to 100 times greater than those in the KO hosts (*p* = 0.0295 by *t* test). This finding is comparable to that for transplanting normal mammary epithelium reported previously [7], which suggested that the mammary stroma of young (6–12 weeks old) 129:*Stat1* ^*−/−*^ mice is less supportive of tumor growth than stroma in age-matched WT hosts.

### Host response

The *Stat1* ^−/−^ tumors exhibited a pronounced local inflammatory response at the interface of tumor cells and surrounding stroma. In contrast to mammary tumors from other GMM models [5], each 129:*Stat1* ^*−/−*^ tumor was surrounded by a thick mantle of host inflammatory cells and fibrosis (Fig. 7, Additional file 2: Figure S9). The inflammation consisted primarily of a nearly equal mixture of F4/80^+^ macrophages and CD3^+^ T lymphocytes in tumor-adjacent stroma. Quantitation of IHC marker-positive cell density in tumor and tumor-associating stroma in spontaneous *Stat1*-null tumors (Additional file 2: Figure S9) indicated tumor-infiltrated F4/80^+^ cells were at a significantly (*p* < 0.0001) lower density (6 cells/mm^2^, SE = 2) than the cellular density in the stroma (2580 cells/mm^2^, SE = 473). CD3^+^ cells were also significantly (*p* < 0.0001) less densely populated in tumors (262 cells/mm^2^, SE = 59) compared with the stroma (3590 cells/mm^2^, SE = 439). Thus, significantly fewer immune cells penetrated the tumor, with more intratumoral infiltration of T lymphocytes than macrophages. In addition, the density of B-cell lymphocytes was significantly (*p* < 0.0001) lower in tumors (34 cells/mm^2^, SE = 6) than in the stroma (381 cells/mm^2^, SE = 64), and the total number of B cells was significantly lower than both macrophages (*p* = 0.0002) and T lymphocytes (*p* = 0.0001).

The microenvironmental effect was further characterized in the grafts where primary tumor tissue was transplanted into mammary glands in WT host or KO host animals. The IHC results showed a predominance of F4/80^+^ macrophages and CD3^+^ T-lymphocytes in the tumor-associated stroma (Fig. 7c–f). The mean density of F4/80^+^ cells in the tumor interstices from a WT host was 19 cells/mm^2^ (SE = 3). By comparison, there was a significantly higher density of F4/80^+^ cells in the surrounding WT stroma (2472 cells/mm^2^, SE = 243). Similarly, transplants of the same primary tumor into the age-matched KO host also showed a predominance of macrophages in the stroma, with only 5 cells/mm^2^ (SE = 2) in the tumor interstices and 2102 cells/mm^2^ (SE = 233) in the stroma. The density of tumor-infiltrated macrophages was significantly higher in the tumors engrafted into a WT host than either the KO host or the spontaneous *Stat1*-null tumors (Additional file 2: Figure S9b). Similarly, CD3^+^ T lymphocytes were found in greater density in the stroma of WT hosts (1990 cells/mm^2^, SE = 218) than in the tumors (463 cells/mm^2^, SE = 54) (*p* < 0.0001). CD3^+^ cell density in KO hosts was significantly lower in both tumors (353 cells/mm^2^, SE = 65) and stroma (1530 cells/mm^2^, SE = 194) than in the WT hosts, but the CD3^+^ cell density was still higher in stroma than in tumors (Additional file 2: Figure S9d). The ratios of tumor-infiltrated immune cells to total cell count in WT, KO host, and spontaneous *Stat1*-null tumors were 8%, 4.5%, and 2.2%, respectively, for CD3^+^ T lymphocytes and 0.77%, 0.24%, and 0.23% for F4/80 macrophages, respectively. In contrast, the cell density of B lymphocytes in stroma was lower in transplants than spontaneous 129:*Stat1* ^*−/−*^ tumors. The density of B lymphocytes was higher in the stroma of WT hosts than the tumors, but there was no significant difference between the tumor and stroma in KO hosts (Additional file 2: Figure S9f). Thus, as with the primary tumors, the host immune cells, especially macrophages, rarely invaded the interstices of solid tumors. This pattern of immune cell reaction to tumors has been described as the “excluded infiltrate” phenotype, in contrast to tumors with a sparse or absent immune reaction, as well as those with an intratumoral “inflamed” phenotype [39].

### Macrophage migration assays

Given the abundance of macrophages in the tumor-associated stroma (Fig. 7, Additional file 2: Figure S9), we reasoned that *Stat1*-null tumors might secrete macrophage chemoattractants. To test this possibility, migration assays with the RAW264.7 macrophage cell line were performed using CM from (1) the *Stat1* ^−/−^ tumor cell line SSM2, (2) the *Stat1* ^+/+^ normal MECs (EpH4, EpH3) and the polyoma middle T-induced mammary tumor cell line Met1, and (4) the Neu-induced mammary tumor cell line NDL. Migration of GFP-H2B-transduced macrophages on 2D plastic was monitored using time-lapse imaging to record track length and time of the mobile cell nuclei.

**Fig. 8:**
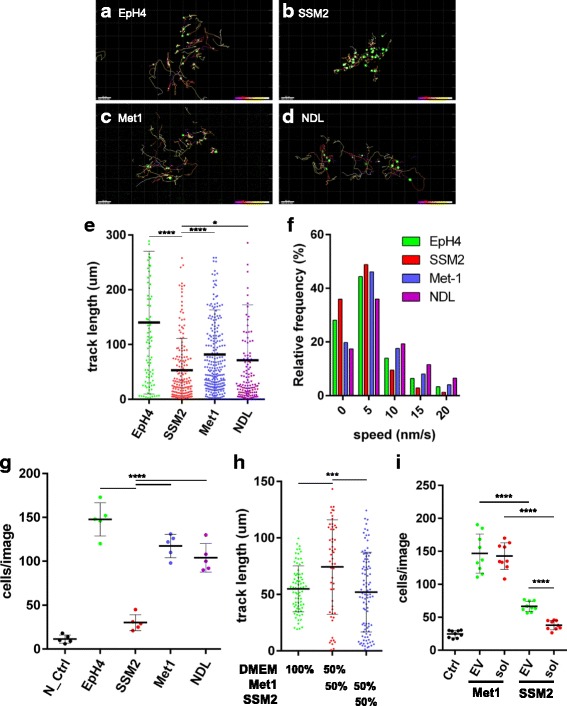
*Stat1*-null cell line secretes macrophage migration stimulatory and inhibitory factors. Green fluorescent protein (GFP)-H2B-transduced RAW264.7 macrophage cells (RAW264.7/GFP-H2B) treated with CM of (**a**) EpH4, (**b**) SSM2, (**c**) Met1, or (**d**) NDL cells were analyzed using time-lapse imaging. **a**–**d** Images are GFP-H2B (*green*) in RAW264.7 cells and tracks of cells migrating. Color legend (*bottom right corners* of **a**–**d**) indicates the time course and reflects the position at a certain time point. Cell migration was evaluated on (**e**) track length and (**f**) migration speed at each time-lapse interval. **g** Graph shows the result of the Transwell migration assay with negative control (N_Ctrl; DMEM) and CM isolated from EpH4, SSM2, Met1, and NDL cells. **h** SSM2-CM showed inhibitory activity on macrophage migration activated by Met1-CM. The effect of conditioned medium of DMEM, Met1/DMEM (50%/50%), and Met1/SSM2 (50%/50%) on RAW264.7/GFP-H2B migration measured by time-lapse imaging is shown. **i** RAW264.7 migration activity in response to either control (Ctrl; DMEM), the extracellular vesicle (EV)-rich fraction, or the soluble fraction (Sol) of Met1- and SSM2-CM measured using the Transwell migration assay. The result indicates that the EV-rich fraction has a stimulatory activity on macrophage migration. Data are mean ± SE. ****p < 0.0001, ***p < 0.001, *p < 0.05. *ns* Not significant (*t* test)

Macrophages exposed to CM from the *Stat1*-null tumor cell line SSM2, compared with CM from the other three cell lines, had shorter track lengths for each time-lapse interval and moved more slowly (Fig. 8a–f). Transwell migration assays also confirmed there was less migration by RAW264.7 cells exposed to SSM2-CM than by the other three cell lines (Fig. 8g). These experiments suggest that *Stat1*-null cells either secrete less chemoattractant or secrete factors that inhibit macrophage migration.

To distinguish between these and other possibilities, SSM2-CM was mixed with the markedly stronger chemoattractive Met1-CM, and we compared this mixture with DMEM/Met1 using the cell-tracking technique (Fig. 8h). Whereas the mixture of Met1-CM and DMEM (50%/50%) resulted in significant movement of RAW264.7 cells, the mixture of Met1-CM and SSM2-CM (50%/50%) reduced the track length of Met1-CM to the level of DMEM alone (Fig. 8g). This supports the notion that SSM2-CM inhibits macrophage migration. This finding is also consistent with the observation of macrophage exclusion from the *Stat1*-KO tumors.

The above results did not, however, explain the dense accumulation of macrophages observed in the stroma surrounding tumors (Fig. 7, Additional file 2: Figure S9), raising the possibility that 129:*Stat1* ^−/−^ cells might secrete factors that can both stimulate and inhibit macrophage migration. Recent studies have shown that EVs/exosomes can stimulate macrophage migration [40], which could explain the attraction of macrophages to the tumor-associated stroma. To test this possibility, Met1-CM and SSM2-CM were fractionated into EV-rich and soluble factor-rich portions using ultracentrifugation. The effects of these fractions on RAW264.7 cell migration were subsequently tested using the Transwell migration assay. Both the supernatant (soluble portion) and EV-rich fraction of Met1-CM induced higher levels of macrophage migration than the same fractions from SSM2-CM (Fig. 8i). Interestingly, the SSM2 EV-rich fraction induced more macrophages to migrate than the soluble fraction of SSM2-CM in the Transwell migration assay (Fig. 8i). The migration is notably comparable to that of the undiluted, noncentrifuged SSM2 media (Fig. 8g). This suggests that factors secreted as EVs from *Stat1*-null tumors are more attractive to macrophages than factors in the soluble portion that inhibit macrophage migration. These could prevent macrophages from infiltrating the tumor, helping to create the excluded infiltrate phenotype.

## Discussion

In the 129:*Stat1* ^−/−^ model of breast cancer, germline deletion results in host, stromal, and epithelial changes that de-suppress mammary adenocarcinoma [2]. These tumors differ from those arising in mice harboring other Stat1-targeting KO constructs or in other mice that are knocked out for tumor suppressor genes such as *Trp53* or *Brca1*. 129:*Stat1* ^−/−^ tumors share a signature histological and cytological phenotype, with ER^+^, PR^+^, and FoxA1^+^ LOP cells. This degree of tumor homogeneity suggests that the same oncogenic driver leads to tumorigenesis [38]. In our experience, the other tumor suppressor KO models develop a variety of tumor types, suggesting they involve a diversity of oncogenic drivers [5].

Consideration of oncogenesis in this *Stat1* ^−/−^ model requires integrating the disease state within the context of animal aging. We report that mammary tumorigenesis in 129:*Stat1* ^−/−^ mice is associated with important pathological features that make this model comparable to human luminal breast cancers. These include ductal origin, topographical asymmetry, age-related ablation of the ovaries, endocrine regulation, host immune response, and tumor-secreted factors. Taken together, this model uniquely reflects host-tumor relationships in an aging host during mammary tumorigenesis.

### Epithelial fitness: aging and pregnancy

The majority of human breast cancers occur in older postmenopausal women. Ironically, this is the first mouse model that directly relates age to mammary tumorigenesis. The prolonged latency and acceleration of tumorigenesis following pregnancy are two key features of tumorigenesis in 129:*Stat1* ^−/−^ females. Because the STAT1 deficiency is a germline mutation, the emergence of tumors requires secondary mutations and/or other adaptations within the microenvironment. The prolonged latency supports the “adaptive oncogenesis” theory, which postulates that changes in the host microenvironment facilitate the expansion of preexisting mutant populations [41]. Altered “fitness” then favors the emergence of specific subsets of mutated cells adapted for the new, aging environment. Although well-documented in the hematopoietic system [42, 43], the aging microenvironment in breast cancer has only recently been reviewed in detail [8].

Although “aging” in mice may vary with strain, animals aged 58 weeks or older can generally be considered “old” [44]. Mice in the present study were aged 30 to 120 weeks (2.3 years), with 38 being older than 70 weeks, which enabled documentation of numerous age-related pathological changes. The most dramatic nonmammary morphological changes in the aging 129:*Stat1* ^*−/−*^ mice occurred in the uterus and ovaries. Whereas rete cysts of the ovary were found in aging 129:*Stat1* ^*−/−*^ animals, they were present in both tumor-free and tumor-bearing animals and thus cannot be considered causal. Nonetheless, they provide direct morphological evidence of age-related changes in the ovary reflecting endocrine changes in an age-related environmental milieu.

Pregnancy accelerated tumorigenesis in 129:*Stat1* ^*−/−*^ females and reduced the median tumor onset from 91 weeks of age in nulliparous females to 78 weeks. Studies of mouse mammary tumors in virus-induced and GMM models have consistently shown that pregnancy accelerates tumorigenesis. This phenomenon has generally been ascribed to the hormone responsiveness of *MMTV-LTR* and/or other mammary-specific promoters. Our previous mammary development experiments demonstrated that the mammary fat pads of young 129:*Stat1* ^−/−^ mice were deficient in locally derived stromal cytokines and did not support optimal growth of the normal and neoplastic mammary epithelium, but they could be reversed by administering exogenous PRL and progesterone [2]. In the present study, we show that transplant of primary tumors into young nulliparous 129:*Stat1* ^*−/−*^ female hosts resulted in much slower growth onset than in age-matched WT hosts (Additional file 2: Figure S8).

PRL signaling appears to be a critical molecular pathway in the etiology of impaired mammary gland development and tumorigenesis in 129:*Stat1* ^−/−^ mice, in keeping with the demonstration that heterozygous truncating PRLR mutations arise in invasive tumors and some of the MIN lesions [17]. Our finding that some of the intraductal proliferation of the FoxA1^+^/ER^+^ LOP cells and areas of MIN had nontruncating single-nucleotide variants for the PRLR suggests that these variants might be responsible for a weaker, possibly nonprogressing proliferation.

These data also highlight the potential relationship between PRLR and ER expression in mammary cancer. In the mammary glands of ovariectomized mice, exogenous PRL suppresses estrogen- and progesterone-induced proliferation [45], similar to findings in transgenic mice that overexpress local PRL [46]. Estrogen and PRL synergistically evoke epithelial proliferation in the mammary glands of pigs [47], whereas overexpression of local PRL in the mammary glands of mice leads to ER-positive mammary tumors [48], and PRL induces ER expression in cultured breast cancer cells [49]. However, cooperation between these two hormones to effect breast cancer cell proliferation varies in accordance with the physical properties of the cell’s microenvironment [50], where the combination of estrogen and PRL enhances breast cancer cell proliferation in stiff collagen but not in low-density collagen. Reciprocal ER and PRLR signaling in a fibrotic microenvironment around *Stat1*-null tumors may drive LOP cell proliferation.

### Ductal progenitor cells, oval cells, and ducts

129:*Stat1* ^−/−^ mammary carcinomas are exclusively FoxA1^+^ as well as ER^+^ and PR^+^. Recent evidence indicates that FoxA1, a “pioneer transcription factor” [51–53], is necessary for ER expression [53–55] and for branching morphogenesis in the mammary glands by maintaining ductal and alveolar luminal cell lineages from basal stem/progenitor cells [54]. ER^+^, PR^+^, and FoxA1^+^ MECs belong to a class of “hormone-sensing” (HS) MECs thought to be directly involved in mammary differentiation and in some human breast cancers [56]. These cells stand out in H&E-stained mammary ducts as morphologically unique, pale, basaloid or suprabasal cells (Additional file 2: Figure S5). They are abundant in mammary glands from young mice but are sparse in older mice (Additional file 2: Figure S5a–c). They resemble the oval clear cells in the mouse mammary gland described by Smith and Medina as “committed progenitor cells” [57, 58], as well as the suprabasal clear cells described in the human mammary gland [59–62].

FoxA1 is expressed in ductal progenitor cells [63, 64]. The location and distribution of the morphologically unique FoxA1^+^ LOP cells identified in the present study are consistent with the notion that LOP cells represent a neoplastic form of suprabasal ductal HS-MEC progenitors [56]. The presence of FoxA1^+^/ER^+^/PR^+^ LOP cells in the 129:*Stat1* ^*−/−*^ mammary ducts and neoplasms is also consistent with a ductal, rather than alveolar, origin of the tumors (Figs. 4, 5, and 6).

The LOP cells in 129:*Stat1* ^−/−^ mammary glands are larger and more pleomorphic than either the FoxA1^+^ cells in normal ducts or the oval clear cells described by Smith and Medina [57, 58]. Individual LOP cells are present in some ducts associated with MIN and their malignant counterparts. The LOP cells also accumulate in tumor-free mammary glands and ducts as small, atypical lesions. As these lesions become larger, the LOP cells form small clumps along the involved mammary duct (Fig. 3), perhaps as abortive side branches (Figs. 4 and 6). This phenomenon is reminiscent of the aberrant branching morphogenesis in the developing 129:*Stat1* ^−/−^ mammary gland [7], where excess abortive side buds are also present in other GMM models [65].

### The sick lobe

The human terminal duct lobular unit (TDLU) has been identified as the most likely site of origin of most human breast cancers [66–69]. Computer-assisted 3D reconstructions of the mammary tree of human breasts demonstrated that ductal carcinoma in situ (DCIS) is limited to a single lobe [70, 71], with “multifocality” confined to foci within a single “sick lobe” [72]. The “sick lobe hypothesis” is also supported by the observation that recurrent invasive cancers and DCIS are found at the site of the prior biopsy or excision that showed DCIS [73]. In contrast, the quiescent and nulliparous mouse mammary glands do not have a specific counterpart to the human TDLU, but they do have HS progenitor cells immune-positive for FoxA1 that are involved in ductal development [54]. As demonstrated here, potentially neoplastic Stat1-null progenitor LOP cells, marked by FoxA1, appear to be topographically limited to a single branch of the mouse mammary tree (Fig. 5).

This “branch” (lobe)-restricted oncogenesis has not been described in other GMM or other mouse models of breast cancer. Carcinogenesis in most GMM models uses alveolar MEC-specific promoters and strong oncogenic transgenes that potentially activate oncogenic pathways in all MECs, resulting in scattered neoplastic foci in all branches of any given mammary gland. By contrast, the 129:*Stat1* ^−/−^ mouse appears to model relatively rare oncogenic events at the level of primary branches of the mammary tree originating in the ducts rather than the alveoli/TDLU. Thus, 129:*Stat1* ^−/−^ tumorigenesis is a unique mouse model of human breast cancers originating from ducts within an at-risk clonally related segment/branch of the ductal tree and models the “sick lobe” of humans.

### Tumor cell factors: protumor chemotaxis

Tumor cells influence their environment by paracrine-secreted and cell surface factors [23]. The host immune response to the primary MIN, tumors, and transplants was more intense and extensive than we observed in other GMM models without specific immunostimulatory interventions. Immune reactions to human cancers have been broadly categorized into “immune desert” with little or no immune cell infiltration, “inflamed” with abundant immune cell infiltration into tumor, and “immune excluded” where immune cells accumulate around the periphery of the tumor without infiltration [39]. The 129:*Stat1* ^−/−^ tumors exhibit this “immune excluded” phenotype, whereas tumors resulting from activation of the ErbB2 or Wnt pathways have a scant host response, more like the “immune desert” phenotype [38, 74, 75]. In addition to connective tissue, a marked cellular response in *Stat1* ^−/−^ neoplasms included macrophage and T-cell invasion and a scattering of granulocytic cells such as neutrophils and mast cells. Notably, macrophages have been implicated in the fibrotic response to cancer [40].

On the basis of the observation of a high tumor “excluded infiltrate” mononuclear density [39], we postulated that the *Stat1*-null tumor cells might mediate the host immune response through release of cytokines and other growth factors. Therefore, the *Stat1-*null tumor cell line SSM2 was used to study macrophage migration [7]. Our cell culture experiments suggest that *Stat1*-null tumor cells secrete both macrophage migration-promoting and macrophage migration-inhibiting factors, though we cannot distinguish between a direct and indirect microenvironmental effect. Nevertheless, our data show that chemokine secretions from *Stat1* ^−/−^ tumor cells have dual effects that also help to explain the attraction but lack of penetrance of macrophages in vivo. Macrophages are known to have both tumor-suppressive and tumor-promoting capacity, depending on the context. For example, macrophages can induce fibrosis, contributing to the stiffened tumor-promoting stroma. Soluble inhibitory activity, meanwhile, prevents tumor-suppressive macrophage migration into the tumor cell interstices. This chemokine secretion from *Stat1* ^−/−^ tumor cells is corroborated by the tissue localization and histopathology.

### A model for tumor progression

**Fig. 9:**
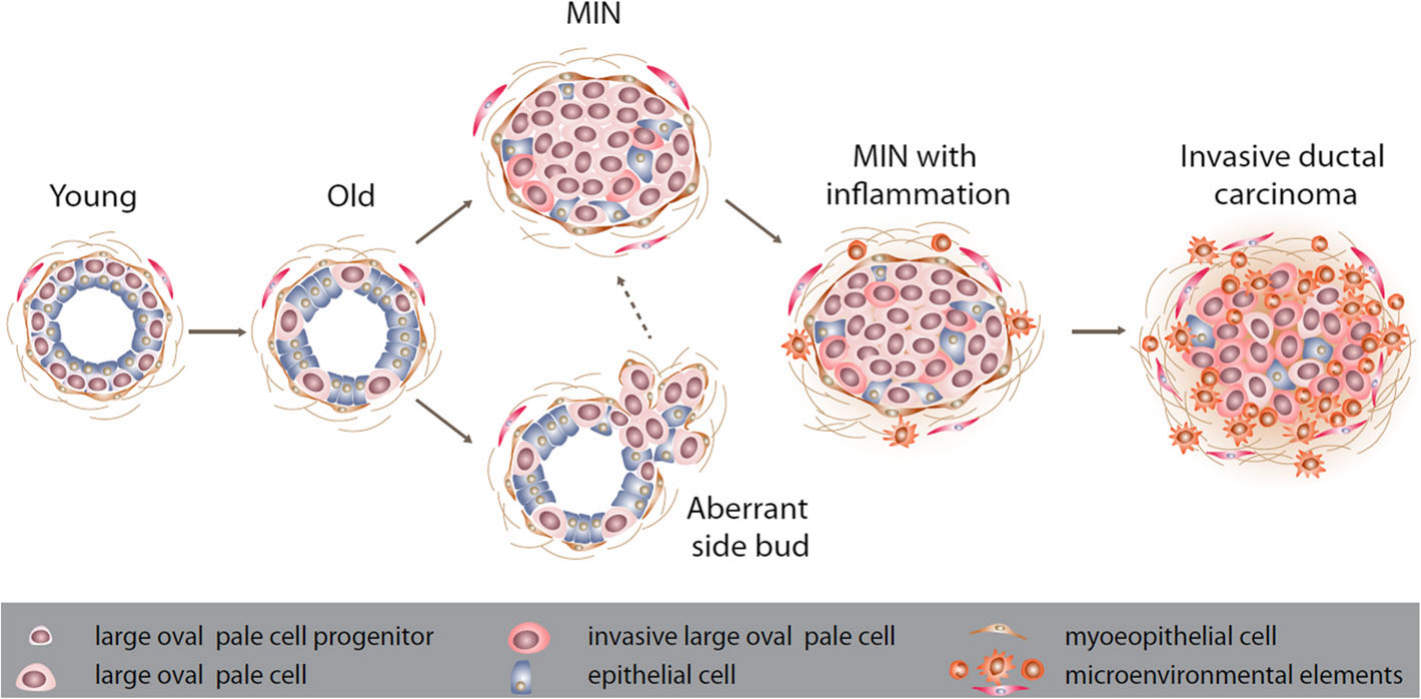
Schematic model of neoplastic progression in 129:*Stat1* ^*−/−*^ tumorigenesis. On the basis of data presented here, a model of neoplastic progression can be proposed. 129:*Stat1* ^*−/−*^ renders (1) a subpopulation of Forkhead box A1-committed progenitor cells (large oval pale [LOP] cells) susceptible to oncogenesis. On the basis of limited distribution of LOP cells within the ductal tree, this tumor progression occurs after the beginning of ductal morphogenesis. The LOP cells have a selective advantage in the 129:*Stat1* ^*−/−*^ cytokine-poor environment. (2) With the onset of adulthood and aging, continued proliferation of a subset of LOP cells gains further selective advantage, forming abortive (aberrant) side buds in some contexts and (3) focal dysplastic proliferation with loss of cell polarity, identified as a mammary intraepithelial neoplasia (MIN). The earliest MIN do not induce a significant host response, exhibit nontruncating prolactin receptor (PRLR) mutations, and generally have a morphologically identifiable basement membrane and myoepithelial layer. (4) However, with further adaptive changes, the MIN LOP cells attract a pronounced protumor host response of macrophages, T lymphocytes, and granulocytes, including mast cells and fibrotic extracellular matrices (microenvironmental elements). (5) The basement membrane and myoepithelial cells become disorganized and disintegrate, with progression of MIN to invasive ductal carcinoma with truncated PRLR mutations

Our observations suggest that *Stat1*-null LOP cells, combined with the age-related changes in the tumor microenvironment, contribute to the development of a malignant neoplasm (Fig. 9). In this model, hormonal and microenvironmental factors induce the expansion of a FoxA1^+^ progenitor population. Further expansion and transformation are the result of a rare event, often a somatic truncating PRLR mutation, that is stochastically favored by the increase in the susceptible progenitor population seen with age. The increase in these progenitors is also associated with aberrant side budding and cysts originating midduct. Progression to invasive carcinoma is preceded by a marked stromal response, suggesting that a critical mass of intraductal cells secreting chemokines is required to create a permissive/tumor-promoting microenvironment.

## Conclusions

The incidence of human breast cancer increases with age, and luminal ER^+^ breast cancers are most frequent in this population. Although luminal breast cancers typically respond to hormone therapy and carry a better prognosis than other subtypes, this subtype accounts for the highest mortality in all age groups. Mouse models of this phenotype are rare, perhaps due to the difficulty in targeting the cell of origin for these cancers. Both “basal” and “luminal” phenotype tumors in GMM models and in human disease appear to arise from luminal progenitors [76, 77], whereas different cells of origin give rise to different tumor types [78]. Most GMM models with expression of transgenes under the control of hormone-responsive promoters such as *MMTV-LTR* or *Wap* generally yield ER-negative tumors primarily located in lobuloalveolar units that are presumably composed of alveolar MECs [1, 79]. Recent work using promoters from MEC progenitors, such as *Lrg5*, produce multilineage tumors [80]. In contrast to those studies, the 129:*Stat1* ^*−/−*^ neoplastic LOP cells express ER and FoxA1, as well as STAT3, STAT5, PR, a truncating PRLR mutation, and downstream elements such as JAK2 and SOCS1 [16, 17]. As a result, a different type of cell, the hormone-sensitive LOP cell, has emerged as a cell of origin, with the resulting neoplasms originating from this ductal progenitor cell. Segmental expansion of the FoxA1^+^ LOP cell population occurs with advanced age and confers an increased risk of this ER^+^ phenotype cancer. In addition, the model, in either the native 129:*Stat1* ^−/−^ mouse or transplanted into WT mice, shows a consistent immune reaction resistant to tumor immune cell infiltration, a phenotype also common in human cancers. Thus, 129:*Stat1* ^−/−^ is a unique model for studying the critical origins and risk reduction strategies in age-related ER^+^ breast cancer in addition to preclinical trials of hormone and targeted therapies as well as immunotherapies.

## Additional files

Additional file 1: Tables S1. Tumor Topography and **S2. ** Tumor incidence in 129: *Stat1* ^-/-^ female mice. (PDF 90 kb)

Additional file 2: Figures S1. Milky ducts in 129: *Stat1* ^-/-^ mammary gland from nulliparous mouse. **Figure S2.** The histological types of MIN. **Figure S3.** LOP cells in 129: *Stat1* ^-/-^ mammary gland. **Figure S4.** Keratin expression in MIN. **Figure S5.** FoxA1 positive cells in 129: WT and 129: *Stat1* ^-/-^ mice. **Figure S6.** FoxA1+/ER+/PR+ positive cells in *Stat1*-null tumor. **Figure S7.** Invasion and metastasis of 129: *Stat1* ^-/-^ tumor. **Figure S8.** *Stat1*-null mammary fat pads poorly support *Stat1*-null tumor growth. **Figure S9.** Immune cells and type IV collagen rich microenvironment in *Stat1*KO tumor. (PDF 6646 kb)

## Abbreviations

CM: Conditioned medium
Cy: Cyanine
DCIS: Ductal carcinoma in situ
EpCAM: Epithelial cell adhesion molecule
ER: Estrogen receptor
EV: Extracellular vesicle
FoxA1: Forkhead box A1
GFP: Green fluorescent protein
GMM: Genetically modified mouse
H&E: Hematoxylin and eosin
HS: Hormone-sensing
IHC: Immunohistochemistry
JAK2/Jak2: Janus kinase 2
KO: Knockout
KRT: Keratin
LN: Lymph node
LOP: Large oval pale
MEC: Mammary epithelial cell
MIN: Mammary intraepithelial neoplasia
NBF: Neutral buffered formalin
PR: Progesterone receptor
PRL: Prolactin
PRLR: Prolactin receptor
TDLU: Terminal ductal lobular unit
TSA: Tyramide signal amplification
WT: Wild type
