# Supplementary material for: Pathobiology of the 129:Stat1−/− mouse model of human age-related ER-positive breast cancer with an immune infiltrate-excluded phenotype
Source: Breast Cancer Res. 2017 Sep 2;19:102. doi: 10.1186/s13058-017-0892-8 (PMC5581425; doi:10.1186/s13058-017-0892-8)
Supplement: Supplementary file 1 — Tumor Topography and S2. Tumor incidence in 129: Stat1 -/- female mice. (PDF 90 kb) [file 13058_2017_892_MOESM1_ESM.pdf]

Additional File 1 (Tables)

Table S1. Tumor Topography

Age (weeks)	Parity*	Cephalad L	Cephalad R	Caudal L	Caudal R
32	P			1	
48	P	1			
54	P	1			
78	P	1	1		
81	P	1	1		
82	P	1			
106	P		1		1
73	N		1		
80	N		1		
82	N	1			
84	N			1	
90	N		1		
90	N		1		
90	N		1		
90	N		1		
90	N		1		
92	N		1		
97	N		1		
106	N	2			
115	N		1		
	Totals	**8**	**13**	**2**	**1**

Total for left (L) =10; total for right (R) =14

*P=Parous; N=Nulliparous

Table S2. Tumor incidence in 129:*Stat1*$^{-/-}$ Female Mice

Age Range (weeks)	Number of Live Animals	Tumor-Free Deceased	Tumor-Bearing Deceased	Total Deceased	Surviving Fraction	Fraction of Mice at Risk
30-50	52	6	2	8	0.85	0.90
50-59	44	5	1	6	0.73	0.85
60-69	38	0	0	0	0.73	0.85
70-79	38	1	2	3	0.67	0.75
80-89	35	9	6	15	0.38	0.45
90-99	20	8	6	14	0.12	0.15
100-120	6	3	3	6	0.00	0.00
	Totals=	32	20	52		
