# Supplementary material for: Pathobiology of the 129:Stat1−/− mouse model of human age-related ER-positive breast cancer with an immune infiltrate-excluded phenotype
Source: Breast Cancer Res. 2017 Sep 2;19:102. doi: 10.1186/s13058-017-0892-8 (PMC5581425; doi:10.1186/s13058-017-0892-8)
Supplement: Supplementary file 2 — Milky ducts in 129: Stat1 -/- mammary gland from nulliparous mouse. Figure S2. The histological types of MIN. Figure S3. LOP cells in 129: Stat1 -/- mammary gland. Figure S4. Keratin expression in MIN. Figure S5. FoxA1 positive cells in 129: WT and 129: Stat1 -/- mice. Figure S6. FoxA1+/ER+/PR+ positive cells in Stat1-null tumor. Figure S7. Invasion and metastasis of 129: Stat1 -/- tumor. Figure S8. Stat1-null mammary fat pads poorly support Stat1-null tumor growth. Figure S9. Immune cells and type IV collagen rich microenvironment in Stat1KO tumor. (PDF 6646 kb) [file 13058_2017_892_MOESM2_ESM.pdf]

Additional File 2 (Figures)

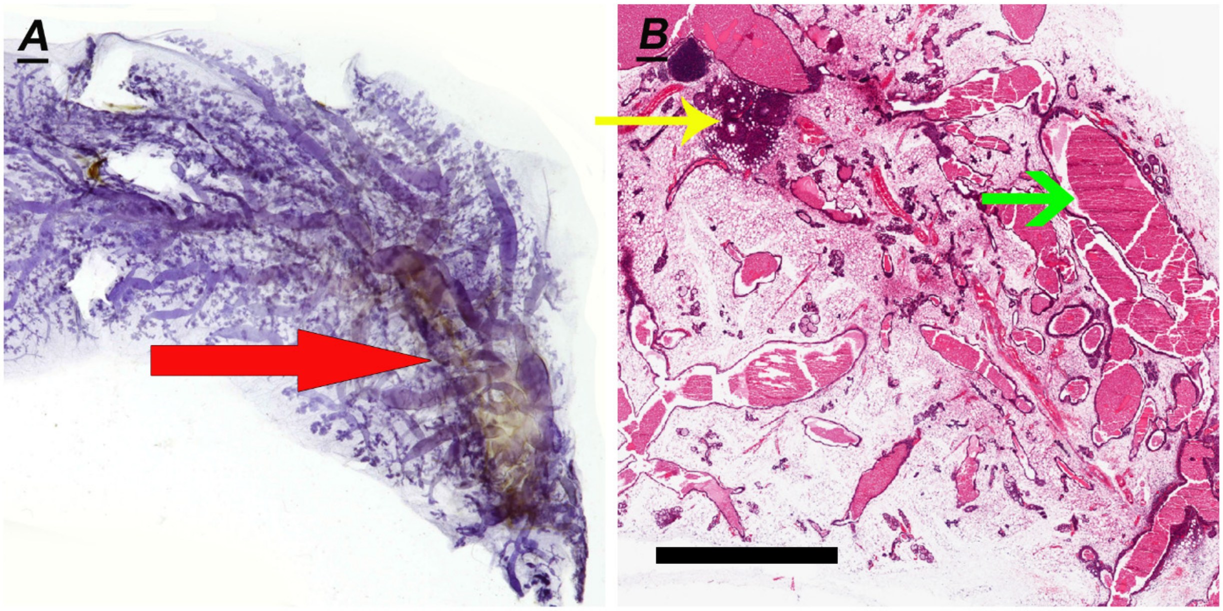

Figure S1. Milky ducts in 129:*Stat1*$^{-/-}$ mammary gland from nulliparous mouse. A) The Hematoxylin stained whole mount from the inguinal mammary fat pad of a nulliparous 106-week-old tumor-bearing 129:*Stat1*$^{-/-}$ female with massively dilated ducts containing white proteinaceous (milky) fluid (red arrow) and diffuse lobuloalveolar hyperplasia. B) The H&E stained microscopic section of the fat pad showing the dilated ducts filled with eosinophilic proteinaceous fluid (green arrow). Although this fat pad did not contain palpable tumors, a small MIN is present (yellow arrow).

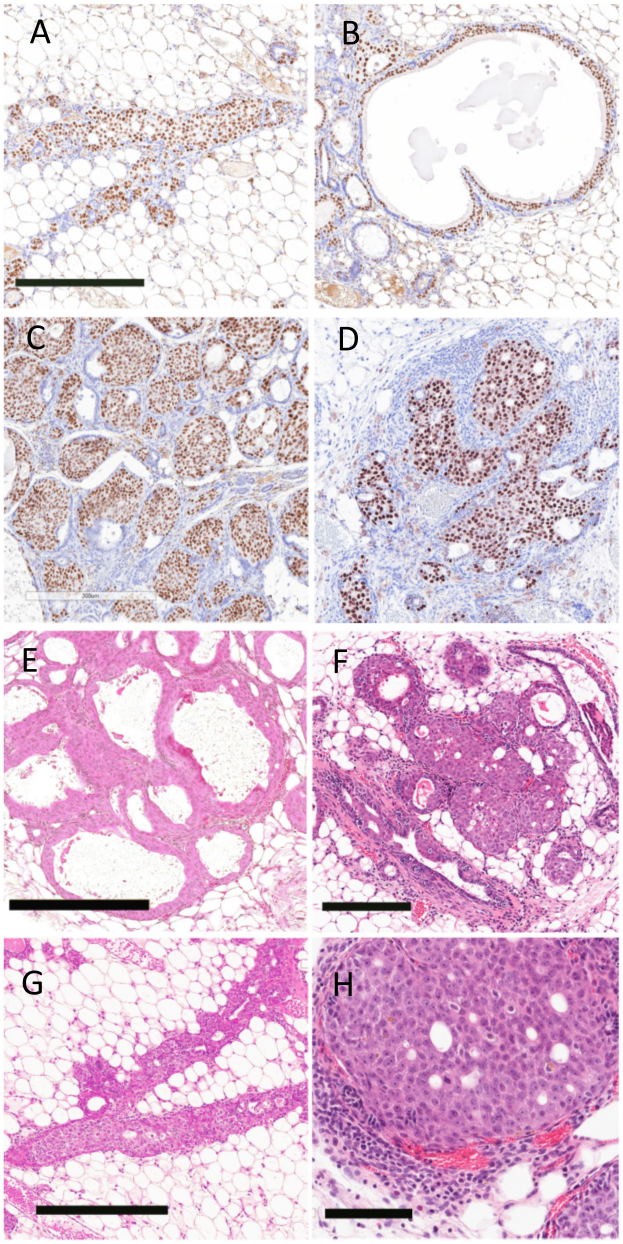

Figure S2. The Histological Types of MIN. (A-H) This panel illustrates the three types of MIN from tumor-free mammary glands from tumor-bearing, 106-week-old nulliparous 129:*Stat1*$^{-/-}$ (A-C and E-G) and 90-week-old tumor bearing 129:*Stat1*$^{-/-}$ females (D and H), This panel illustrates the three types of MIN lesions found in the 129:*Stat1*$^{-/-}$ female stained with FoxA1 and H&E. (B and E) Cystic with expanded luminal cystic space lined by LOP cells. Note the lack of any appreciable host response. Scale Bar = 300 μm. (C and F) Solid Micro-Nodular pattern with small nests of cells in dense connective tissue. Scale Bar = 200 μm. (A-G) Ductal pattern with a branching duct filled with LOP cells. Scale Bar = 300 μm. (D and H) A higher magnification of a solid nodular MIN with dilated vessels and dense round cell infiltrate. Scale Bar = 100 μm.

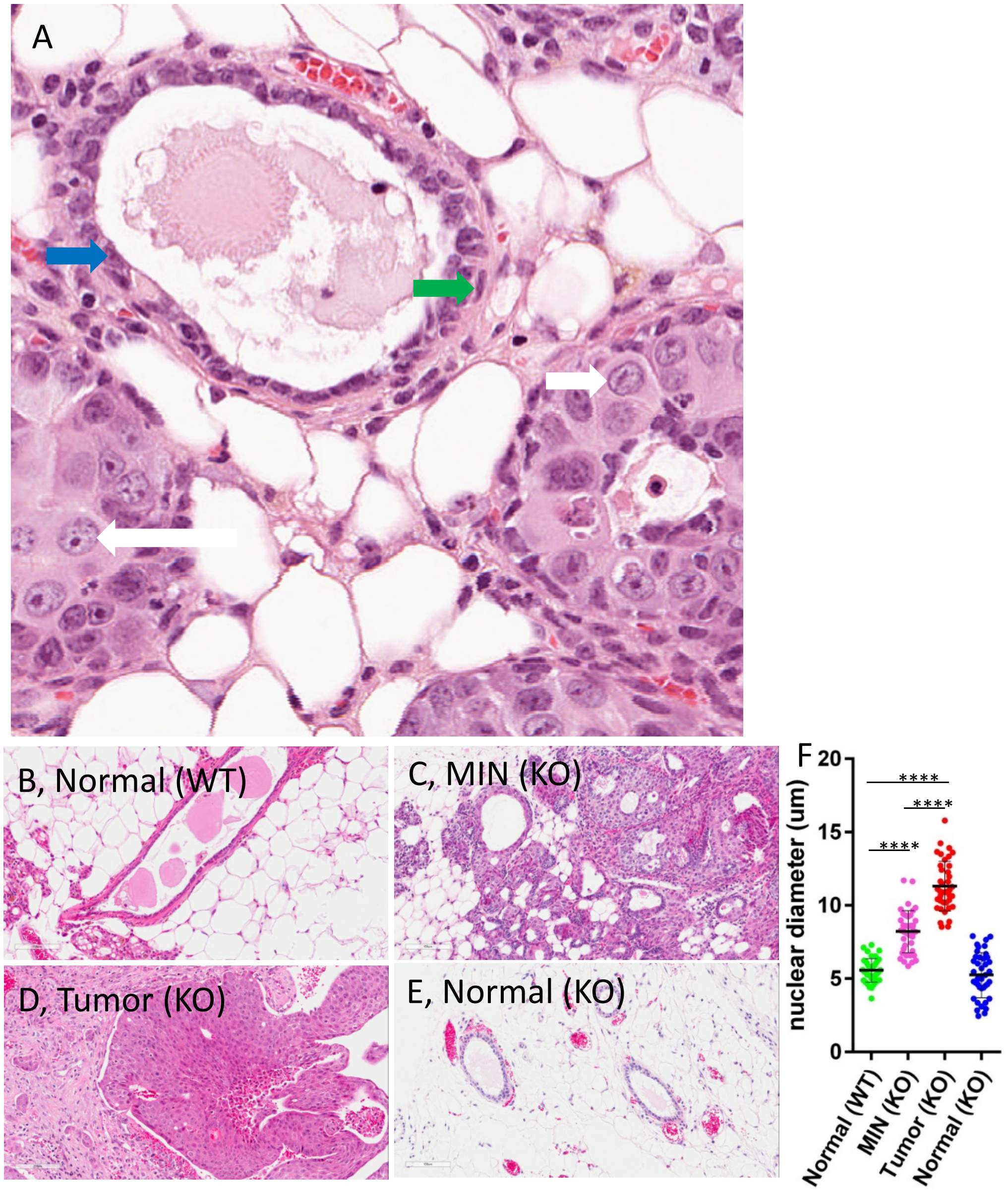

Figure S3. LOP cells in 129:Stat1$^{-/-}$ mammary gland. (A) H&E stained tissue section of 129:*Stat1*$^{-/-}$ mammary gland shows normal ductal luminal cell (blue arrow), basal cell (green) and LOP cells in MIN (white). The MEC in normal duct are smaller and darker staining with smaller nuclei. The LOP cells in MIN have larger nuclei with open chromatin and more abundant, paler staining cytoplasm. The size of LOP cells were compared with normal mature luminal cells in H&E stained tissue sections from (B) 129:WT mammary gland and (C-E) 129:*Stat1*$^{-/-}$ mammary tumors and normal ducts. (F) The graph is showing the measurement of nuclear diameter (longer diameter) in normal cells in WT (green), MIN cells in KO (pink), tumor cells in KO (red) and normal cells in KO (blue). At least 50 cells were measured in each category. Data are mean ± s.e.m. ****P<0.0001 (t-test).

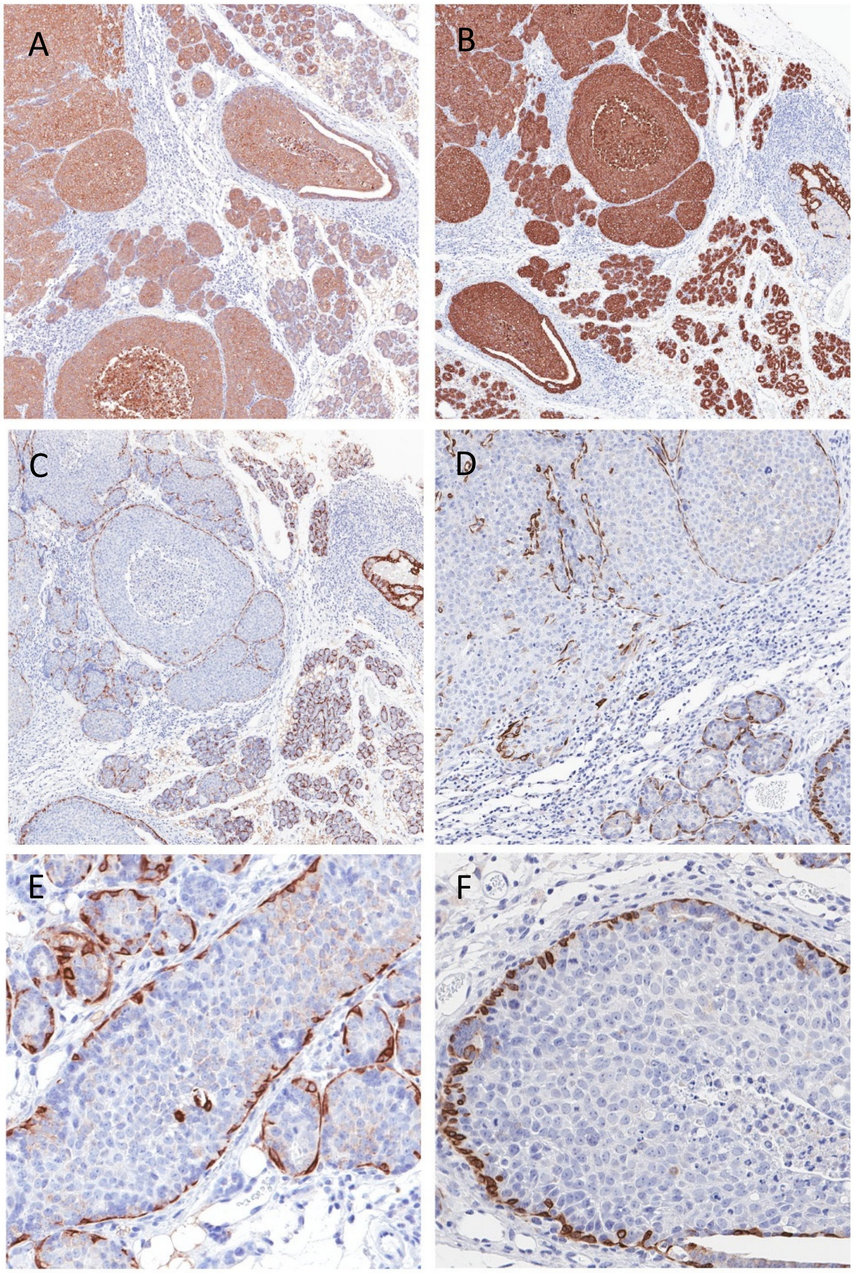

Figure S4. Keratin expression in MIN. This panel illustrates that the KO MIN and Tumors are expressing (A) KRT8/18, (B) KRT19 and (C-F) KRT14. C-F demonstrate that the MIN have a peripheral rim of KRT14 positive myoepithelial cells that disappear as the neoplasm progresses to an invasive malignancy (C and D). The MIN is normally encased in a myoepithelium but scattered luminal cells also stain for KRT14 (E and F). These cells are also FoxA1 positive (see Figure S3) demonstrating that the FoxA1 LOP cells are pluripotential.

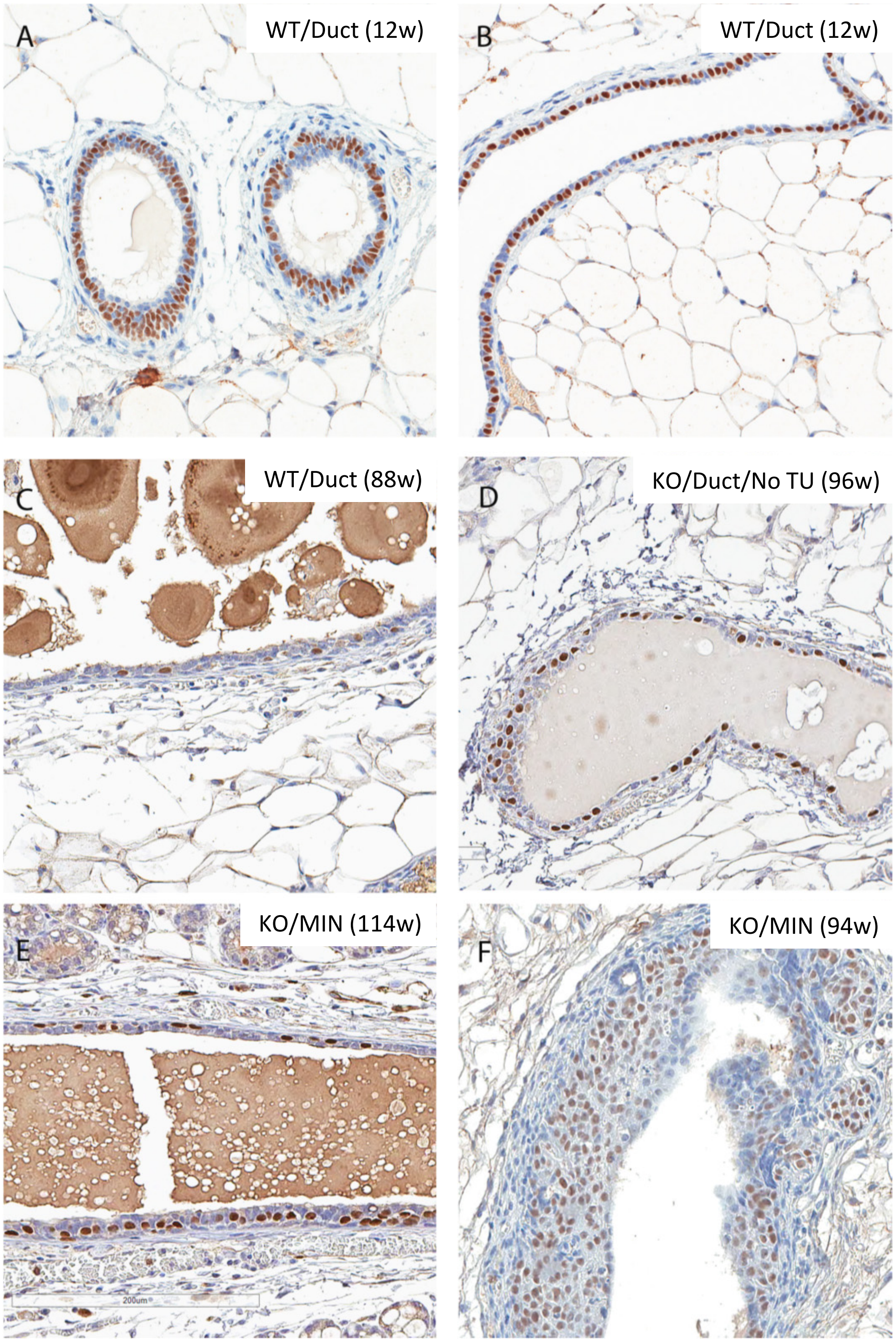

Figure S5. FoxA1 positive cells in 129:WT and 129:*Stat1*$^{-/-}$ mice. Shown images are FoxA1+ in: mammary duct from (A, B) 12-week-old WT mouse, (C) 88-week-old WT, (D) 96-week-old KO with no tumor, (E) 114-week-old KO mouse and (F) MIN in 94-week-old KO mouse.

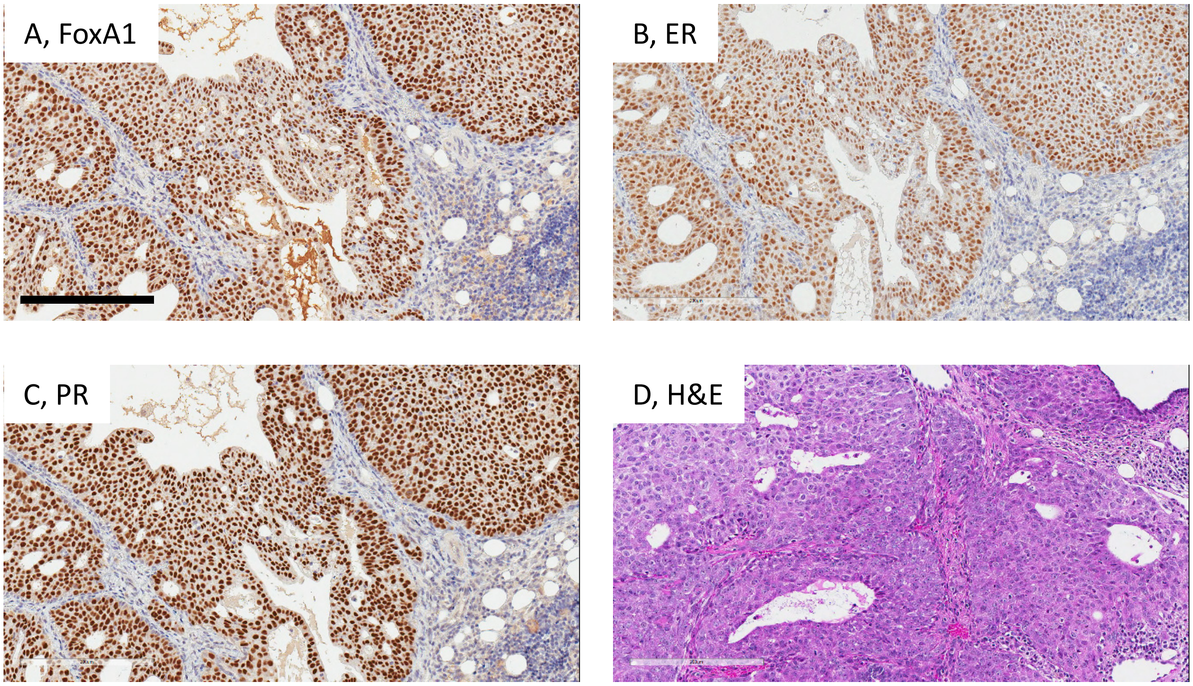

Figure S6. FoxA1+/ER+/PR+ positive cells in *Stat1*-null tumor. Tissue sections of *Stat1*-null primary tumor were immunohistochemically stained with (A) FoxA1, (B) ER and (C) PR. These tumor cells have FoxA1, ER and PR. (D) is indicating a similar area of the tumor stained with H&E. Scale bar=200μm.

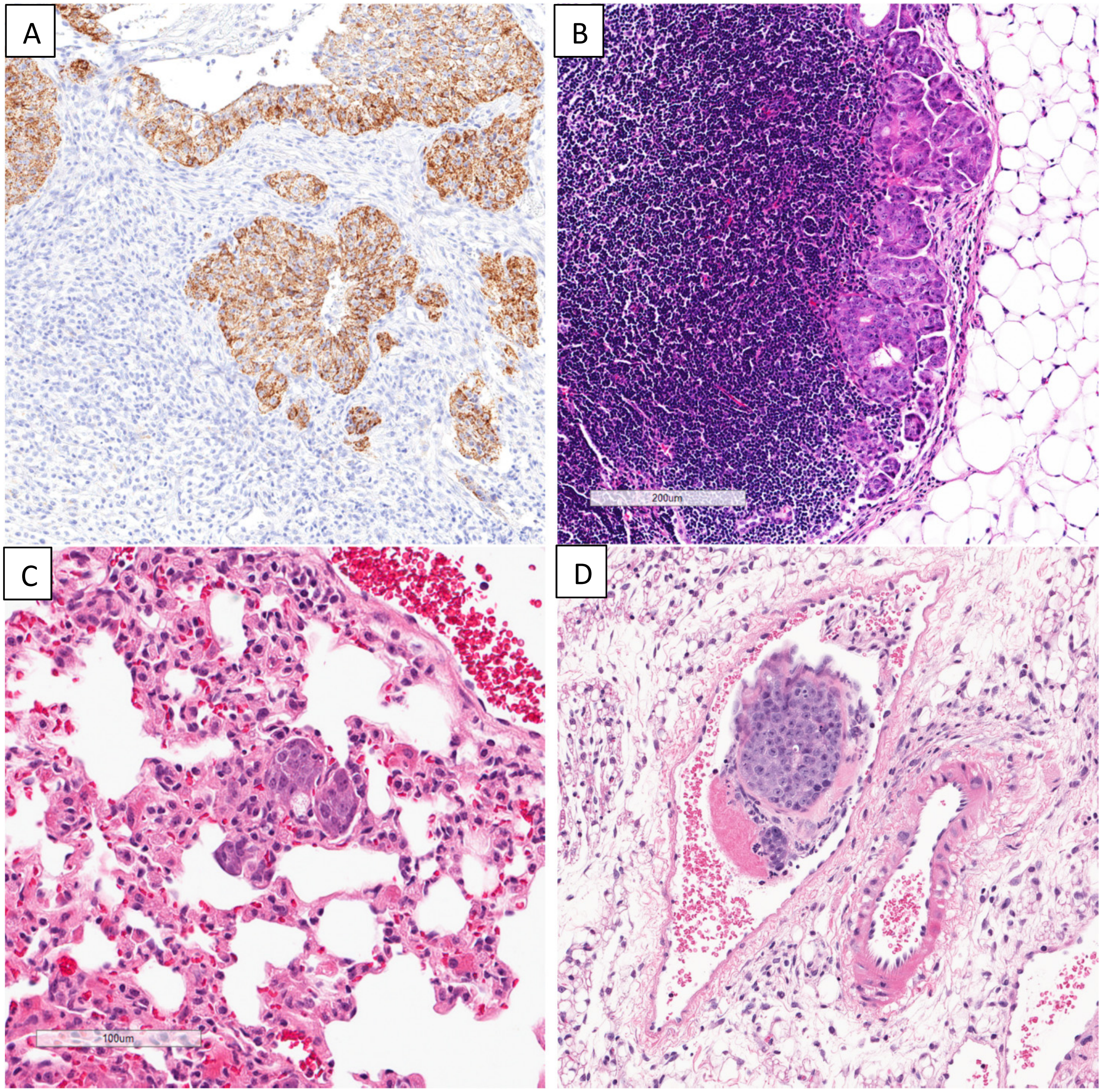

Figure S7. Invasion and metastasis of 129:*Stat1*$^{-/-}$ tumor. Images in panel illustrate (A) tumor cells stained with IHC for Muc-1 invading into a dense host response, (B) H&E stained mammary fat pad lymph node with clusters of metastatic tumor in the sub-capsular sinus, (C) H&E stained lung showing a small tumor cell embolus in alveolar capillary. (D) H&E stained blood vessel in a mammary fat pad with a tumor embolus attached to a fibrin clot.

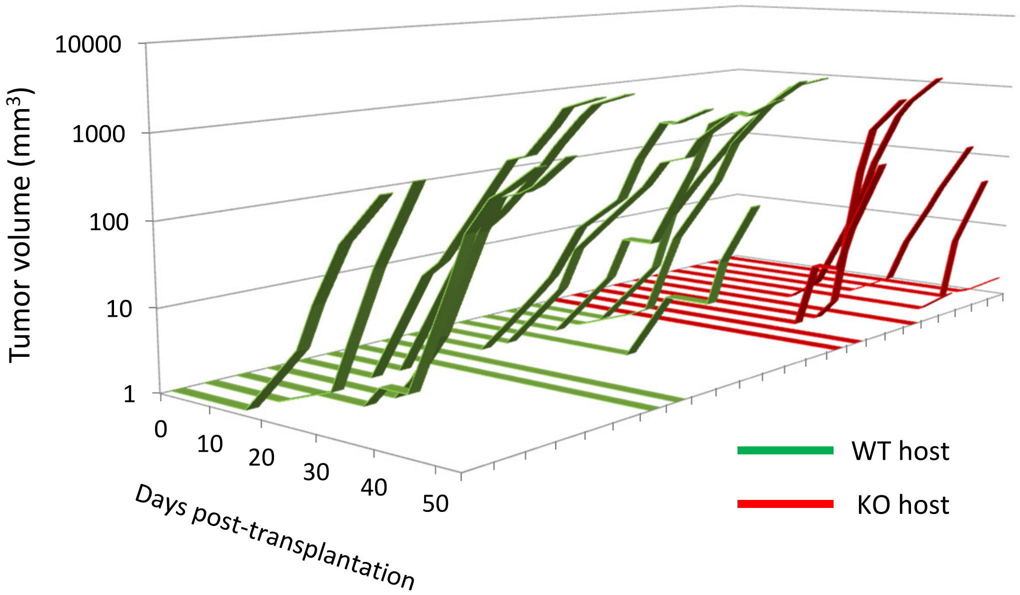

Figure S8. *Stat1*-null mammary fat pads poorly support *Stat1*-null tumor growth. The graph is showing the tumor volumes of transplanted *Stat1*-null tumor in 129:WT (green) and 129:*Stat1*$^{-/-}$(red) mammary fat pads. To analyze a host response to *Stat1*-null tumor, *Stat1*-null primary mammary tumors were transplanted into inguinal mammary fat pads in 129:WT and 129:*Stat1*$^{-/-}$ mice. Seven WT hosts and five KO hosts were analyzed. Tumor growth was measured by caliper twice per week from 14 days after post-operation until day 48. Tumor volume was calculated as (3.14/6)*1.58*(length X Width) ^ (3/2). Whereas *Stat1*-null tumor grew faster in mammary fad pads in 129:WT, the growth was slower in 129:*Stat1*$^{-/-}$.

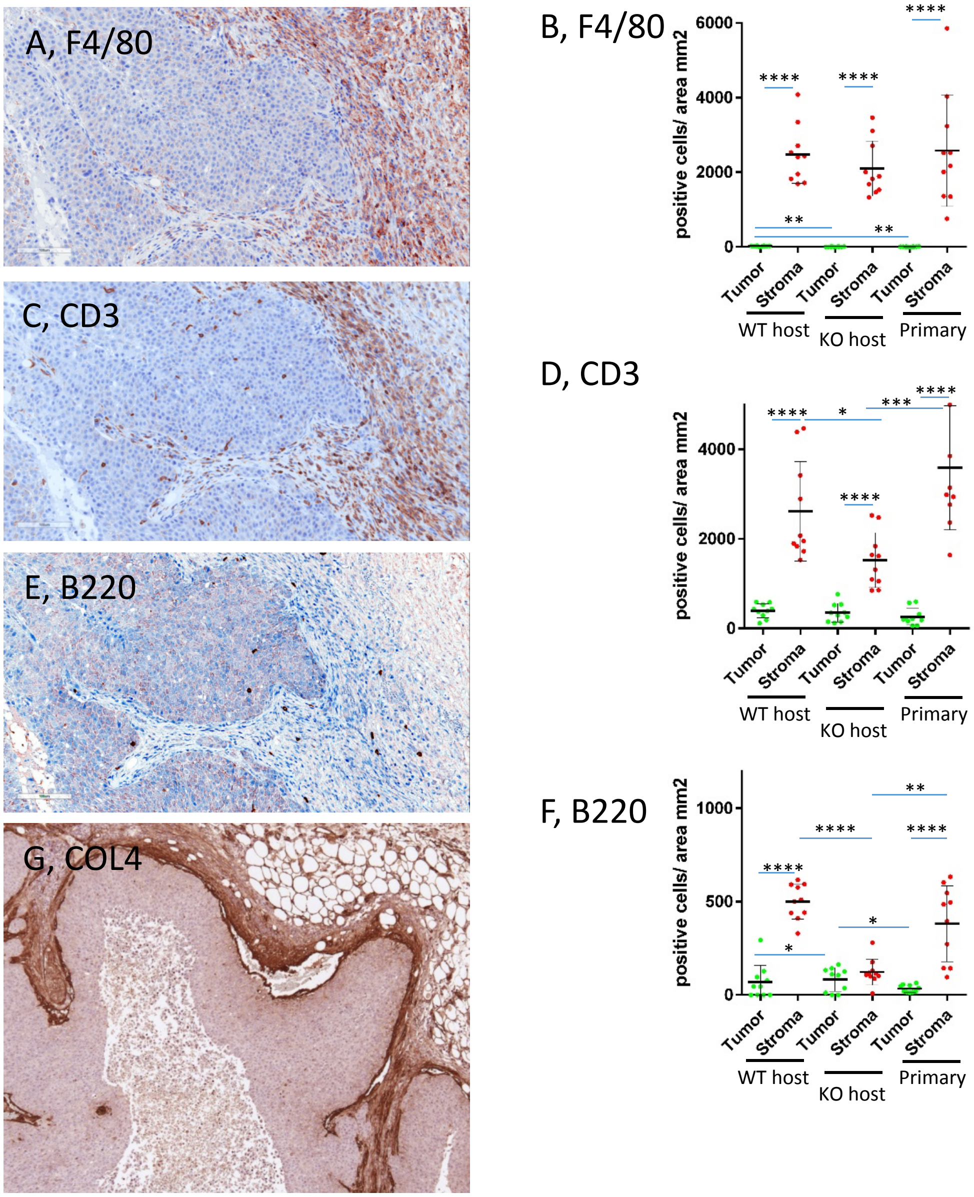

Figure S9. Immune cells and type IV collagen rich microenvironment in *Stat1*KO tumor. Immune cell populations were analyzed in primary *Stat1*-null tumor. Tissue sections were stained with (A) F4/80, (C) CD3 and (E) B220 to detect macrophages, T-lymphocytes and B-cells, respectively. (B, D and F) The distribution of marker positive cells were analyzed in tumor and stroma from 8 images. Graphs are indicating the cell densities of (B) F4/80, (D) CD3 and (F) B220 positive cells. Data are mean±s.e.m. ****P<0.0001, ***P<0.001, **P<0.01, *P<0.05 (t-test). (G) IHC for type IV collagen (COL4) is shown.
